# *IFIH1* Contributes to M1 Macrophage Polarization in ARDS

**DOI:** 10.3389/fimmu.2020.580838

**Published:** 2021-01-14

**Authors:** Shi Zhang, Cuilin Chu, Zongsheng Wu, Feng Liu, Jianfeng Xie, Yi Yang, Haibo Qiu

**Affiliations:** Jiangsu Provincial Key Laboratory of Critical Care Medicine, Department of Critical Care Medicine, Zhongda Hospital, School of Medicine, Southeast University, Nanjing, China

**Keywords:** acute respiratory distress syndrome, macrophage, M1 polarization, *IFIH1*, *IRF3*

## Abstract

Accumulated evidence has demonstrated that the macrophage phenotypic switch from M0 to M1 is crucial in the initiation of the inflammatory process of acute respiratory distress syndrome (ARDS). Better insight into the molecular control of M1 macrophages in ARDS may identify potential therapeutic targets. In the current study, 36 candidate genes associated with the severity of ARDS and simultaneously involved in M1-polarized macrophages were first screened through a weighted network algorithm on all gene expression profiles from the 26 ARDS patients and empirical Bayes analysis on the gene expression profiles of macrophages. *STAT1, IFIH1, GBP1, IFIT3*, and *IRF1* were subsequently identified as hub genes according to connectivity degree analysis and multiple external validations. Among these candidate genes, *IFIH1* had the strongest connection with ARDS through the RobustRankAggreg algorithm. It was selected as a crucial gene for further investigation. *For in vitro* validation, the RAW264.7 cell line and BMDMs were transfected with sh*IFIH1* lentivirus and plasmid expression vectors of *IFIH1*. Cellular experimental studies further confirmed that *IFIH1* was a novel regulator for promoting M1 macrophage polarization. Moreover, gene set enrichment analysis (GSEA) and *in vitro* validations indicated that *IFIH1* regulated M1 polarization by activating *IRF3*. In addition, previous studies demonstrated that activation of *IFIH1-IRF3* was stimulated by viral RNAs or RNA mimics. Surprisingly, the current study found that LPS could also induce *IFIH1*-*IRF3* activation *via a* MyD88-dependent mechanism. We also found that only *IFIH1* expression without LPS or RNA mimic stimulation could not affect *IRF3* activation and M1 macrophage polarization. These findings were validated on two types of macrophages, RAW264.7 cells and BMDMs, which expanded the knowledge on the inflammatory roles of *IFIH1* and *IRF3*, suggesting *IFIH1* as a potential target for ARDS treatment.

## Introduction

Acute respiratory distress syndrome (ARDS), a common and severe pulmonary complication of critical illness, affects approximately 10%–15% of patients hospitalized in the intensive care unit (ICU) (1, 2). Despite advances in clinical management and basic science, the mortality rate of severe ARDS remains 40%–46% (2, 3). In the last decade, most innovative treatments have failed, and there is an obvious, robust need for better insight into the pathogenesis of ARDS and subsequent emerging therapeutic options (4–10).

Uncontrolled inflammatory responses in the lungs induce perpetuation of lung injury, impacting the severity of ARDS and patient prognosis, and macrophages/monocytes play a causal role in this pathogenesis. During the first hours after injury, alveolar macrophages enhance lung inflammation. Subsequently, substantial numbers of recruited monocytes invade the alveoli during the next 12–24 h. At any point in this initial inflammatory process of ARDS, alveolar macrophages and monocytes can be polarized into pro-inflammatory populations (M1 phenotype). Macrophages with an M1 phenotype further promote lung inflammation through secretion of inflammatory cytokines and chemokines, which consistently activate and recruit immune cells, leading to continuous lung injury (11–15). Therefore, it may be therapeutically valuable to attenuate macrophage polarization into the M1 phenotype in the initial stage of ARDS.

Previous studies on macrophage polarization have identified molecules and signal transduction pathways that contribute to M1 polarization. However, these studies mainly focused on tumors and other diseases, not ARDS (16–30). The molecular control of macrophage polarization involved in the development of ARDS remains largely unknown. Based on this situation, we constructed the ARDS biological specimen bank in 2017, which we have used to screen molecules associated with ARDS. Meanwhile, public high-throughput datasets (the GEO database) provide ample available data for the exploration of molecules involved in M1 macrophage polarization.

In the present study, *IFIH1* was identified as a crucial molecule that might be simultaneously involved in the development of ARDS and M1 polarization by using bioinformatic analysis and experiments. Furthermore, transfected RAW264.7 cell lines and bone marrow-derived macrophages (BMDMs) with *IFIH1* overexpression or knockdown were constructed and utilized to confirm that *IFIH1* is a novel molecular regulator of M1 macrophage polarization. The subsequent bioinformatic analysis and experiments on biological pathways indicated that *IFIH1* regulates M1 macrophage polarization *via IRF3* activation.

In addition, *IFIH1* has been identified to code for melanoma differentiation-associated five protein, an intracellular viral sensor. Previous studies have demonstrated that *IFIH1* is a pathogen pattern recognition receptor that is activated by dsRNA (31–35). Interestingly, the current study first found that lipopolysaccharide (LPS) could also activate *IFIH1* and *IRF3*, which are involved in MyD88-dependent mechanisms. These results could further uncover the molecular networks of inflammatory function on M1 macrophage polarization.

## Materials and Methods

The current study included three steps to explore the crucial molecules and underlying mechanisms involving M1-polarized macrophages in ARDS: (1) screen candidates; (2) confirmation *in vitro*; and (3) elaboration of the mechanism.

### Screen Candidates

To screen crucial genes involved in ARDS and M1-polarized macrophages for further research, we conducted interrelated bioinformatic analysis of a mRNA matrix from ARDS patients and gene expression profiles of macrophages. The mRNA matrix from ARDS patients was built from the biological specimen bank of the Critical Care, Zhongda Hospital. These gene expression profiles of macrophages were screened from the public GEO database.

### ARDS Patients

The current research was performed in accordance with the amended Declaration of Helsinki. The human sample collection was reviewed and approved by the Institutional Ethics Committee of Zhongda Hospital. The ethical documents for human studies can be downloaded in attach files.

#### Peripheral Blood Specimens for All Gene Expression Profiles

To screen candidate genes associated with the severity of ARDS, whole blood was collected from 26 ARDS patients to measure all gene expression profiles *via* the Human mRNA Microarray V4.0 (Arraystar) chip. In this study, critically ill patients admitted *via* the emergency department were enrolled at the time of triage to the ICU. Patients were defined as having ARDS if they met the criteria defined by the Berlin definition of ARDS within 24 h of enrolment in the study (36, 37). The exclusion criteria were as follows: any past history of cancers, hematological or immunological disease, or therapies with chemotherapeutic agents or steroids within 6 months prior to hospitalization.

Whole blood was obtained within 24 h of ICU admission for the isolation of RNA. TRIzol reagent (Life Technologies, USA). was utilized to extract total RNA according to the instructions. After removing rRNA (mRNA-ONLY™ Eukaryotic mRNA Isolation Kit, Epicentre), mRNA was purified from the extracted total RNA. Each sample was amplified and transcribed into fluorescent cRNA along the entire transcript length without 3’ bias using a random priming method (Arraystar Flash RNA Labeling Kit, Arraystar), and the labeled cRNAs were purified with the RNeasy Mini Kit (Qiagen). Furthermore, these labeled cRNAs were hybridized onto the Human mRNA Microarray V4.0 (Arraystar) chip, including 20,730 genes. These chips were scanned using an Agilent G2505C scanner. The densities of fluorescence were calculated according to Agilent Feature Extraction software.

#### BALF Specimens for Hub Gene Validation

To validate whether the hub genes are involved in ARDS, we further detected the mRNA expression of the hub genes in bronchoalveolar lavage fluid (BALF) samples from patients with ARDS (n=6) and control patients without ARDS (n=6) *via* RT-PCR. The control group patients were defined as mechanically ventilated patients without ARDS (postoperative patients), since the BALF specimens were availably obtained in these patients.

Patients were inspected according to a standard approach under local anesthesia using fiberoptic bronchoscopy (FOB). The bronchoscopes were wedged into the lingual lobe or the middle lobe in patients with diffuse pulmonary disease. Furthermore, 100 ml physiological saline was instilled and aspirated immediately three times. The BALF was collected. Then, the BALF specimen was centrifuged at 1000 r/min for 20 min, and the cell pellets were collected. Total RNA was extracted using TRIzol reagents, and mRNA expression was measured by RT-PCR. A t test was performed to confirm whether hub gene expression was significantly upregulated or downregulated in ARDS, with a cut-off value of *P <*0.05.

### LPS-Induced ARDS in Mice for Hub Gene Validation

Similarly, to further validate whether the hub genes are involved in ARDS, the mRNA expression of the hub genes was also measured using RT-PCR in lung tissue homogenates from LPS-induced ARDS mice (n=6) and control mice (n=6). A t test was performed to confirm whether hub gene expression was significantly upregulated or downregulated in ARDS, with a cut-off value of *P <*0.05.

The Committee of Animal Care and Use of Southeast University reviewed and approved the animal experiments in the current study. All animal experiments were carried out in accordance with the Institutional Animal Use and Care Committee. The Laboratory Animal Center (Shanghai, China) provided all C57BL/6 mice (male, aged 6–8 weeks) used in experiments. The mice were maintained on a 12/12-h light/dark cycle at 22–26°C with sterile pellet food and water provided ad libitum. After mice were anaesthetized with an intraperitoneal injection of 5.0% (w/v) pentobarbital sodium (4.0 ml/kg), they were subjected to intratracheal administration of LPS (*E. coli* 0111:B4; Sigma-Aldrich, St. Louis, MO). The sham operations were conducted in similar manners with the same volume of normal saline.

### Screening Candidates *via* Interrelated Bioinformatic Analysis

To screen candidates involved in ARDS and M1-polarized macrophages for further research, 5 steps were implemented in this study.

1) To screen candidate genes associated with the severity of ARDS, weighted correlation network analysis (WGCNA) was performed on all gene expression profiles from the 26 ARDS patients. Briefly, we utilized the WGCNA R package to classify all expressed genes in microarrays into several module eigengenes (MEs) based on coexpression relationships (38). Then, the MEs were compared with the severity of ARDS using Spearman’s correlation corrected for clusters. The genes from modules that were highly associated with the severity of ARDS (the maximum correlation coefficient and *P* < 0.05) were selected for further analysis.

The WGCNA algorithm is a systems biology method for describing the correlation patterns among genes across microarray samples. WGCNA can be used for finding clusters (modules) of highly correlated genes, for summarizing such clusters using the module eigengene or an intramodular hub gene, for relating modules to one another and to external sample traits (using eigengene network methodology), and for calculating module membership measures (38).

The process of WGCNA included three steps: construction of gene-correlation networks, scale-free topology and exploration of links between gene networks and clinical characteristics. Construction of gene-correlation networks was based on the biweight midcorrelation or the Spearman correlation, with the formula S_ij_ =|cor(x_i_, x_j_)|. The aim of scale-free topology is to simplify and optimize the gene-correlation networks. A weighed network adjacency can be defined by raising the coexpression similarity to a power, with the formula a_ij_$=s_{\text{ij}}^{\beta }$. The exploration of links between gene networks and clinical characteristics was based on Pearce correlation or Spearman correlation analysis.

2) To investigate the genes potentially involved in M1 macrophage polarization, we need to screen significantly differentially expressed genes between M1 macrophages and M0 and M2 macrophages (2-fold difference, *FDR*<0.05). Therefore, we searched for all high-throughput experiments that matched terms of macrophage M1 polarization in the GEO database. The exclusion criteria were as follows: a) lack of at least one macrophage polarization phenotype, b) incomplete transcriptome data, and c) inability to obtain macrophage polarization phenotype information for each sample. d) Furthermore, if the macrophages were treated with drugs, siRNA, shRNA, plasmid, or CRISPR, these databases were removed because such high-throughput results were deeply influenced by these interventions. e) In addition, studies that lack robust experiments to validate macrophage polarization will be removed. The robust experiments included western blot, flow cytometry, immunofluorescence, and other experiments for investigating macrophage polarization markers. The flow process of this search is shown in **Figure S1**.

The GSE46903 dataset was used as the macrophage training dataset since this dataset had the largest sample size. The other datasets were used as macrophage validation datasets to further observe the results of analysis on the training dataset.

We utilized the Limma R package to identify differentially expressed genes based on mRNA microarray data and the edgeR R package for screening differentially expressed molecules based on RNA sequencing data. The algorithm of differentially expressed analysis implements an empirical Bayesian approach to estimate gene expression changes using moderated t-tests.

3) Furthermore, to identify the candidate genes simultaneously associated with ARDS and M1 macrophages, a Venn diagram was plotted to capture the overlap of the genes found in (1) and (2).

4) To determine the crucial genes, we performed hub gene analysis through connectivity degree analysis (number of neighbors). First, we plotted the protein-protein interaction (PPI) network based on the STRING website (http://string-db.org/cgi/input.pl). Second, the total connectivity degree of each node in the network was calculated using R to find the genes with the highest connectivity degrees. Genes above the first inflection point of connectivity degree were identified as hub genes (39).

5) The gene with the strongest ARDS-related correlation was identified as a primary candidate for further investigation *in vitro*. To select the gene with the strongest ARDS-related correlation, we utilized the RobustRankAggreg algorithm (RobustRankAggreg R package) to calculate interrelated *P* values based on the *P* values from human and animal experiments. The hub genes were ranked according to interrelated *P* values from minimum to maximum. The gene with the minimum interrelated *P* value and maximum Spearman correlation coefficient was selected as the crucial gene for further research (40).

As the outputs of individual experiments can be rather noisy, it is essential to look for findings that are supported by several pieces of evidence to increase the signal and lessen the fraction of false positive findings. The RobustRankAggreg algorithm aims to obtain the best out of all these alternatives according to integrating their results in an unbiased manner. The RobustRankAggreg algorithm calculated the interrelated *P* value through the formula *P*(r)=min_k=1…n_ β_k,n_(r) (40).

### Confirmation *In Vitro*

As the expression of *IFIH1* is known to be up-regulated in M1 polarized macrophage based on the results of bioinformatics analysis, we asked whether *IFIH1* can affect M1 macrophage polarization. To confirm this possibility, shRNA sequence and plasmid expression vectors for *IFIH1* were introduced to construct stably transfected RAW264.7 cell lines with *IFIH1* overexpression or knockdown, respectively, followed by LPS or Poly(I:C) induction for 24 h. Furthermore, we investigated markers of M1 macrophage polarization and pro-inflammatory cytokines in each group *via* western blotting, flow cytometry, and ELISA. These markers of M1 macrophage polarization included *iNOS* and *CD86*. The pro-inflammatory cytokines included *IL-1β* and *CCL2*.

Considering that RAW264.7 cells intensively proliferate, mouse primary macrophages, bone marrow-derived macrophages (BMDMs), were used as supplementary evidence. We re-conducted all experiments on RAW264.7 cells and BMDMs simultaneously.

### Bone Marrow–Derived Macrophage Isolation, Differentiation, and Identification

C57BL/6 mice (male, aged 8 weeks) were killed by cervical dislocation. Then, femurs and tibias were separated from tissue and sterilized with 75% ethanol for 30 min. The bones were cut from both ends and flushed with medium using a 10-ml syringe. Furthermore, these cell media were centrifuged at 1000 rpm and resuspended in new medium. The isolated cells were cultured in DMEM containing 10% FBS and 20 ng/ml M-CSF for 7 days to obtain adherent cells (BMDMs). Flow cytometry was used to detect *F4/80* (macrophage marker) for the identification of BMDMs.

### Cell Culture and Reagent Treatment

The Cell Resource Center of the Shanghai Institutes for Biological Sciences (Chinese Academy of Science) provided us with the murine-derived macrophage cell line RAW264.7. RAW264.7 cells and BMDMs were cultured in Dulbecco’s modified medium (DMEM; Wisent Biotechnology, Nanjing, China) containing 10% fetal bovine serum (FBS, Coring, Australia), 100 IU/ml penicillin, and 100 μg/ml streptomycin at 37°C in a humidified atmosphere with 5% CO2.

It has been robustly demonstrated that *IFIH1* and the *RIG-I* pathway has robustly demonstrated that Poly(I:C) is the activator of *IFIH1* and *RIG-I*. In addition, LPS has been repeatedly confirmed as the classical activator for M1 macrophage polarization. Bacterial lipopolysaccharides have been robustly identified as crucial pathogenic factors of ARDS and are widely used to construct ARDS animal models inflammatory cell models. In consideration of these factors, Poly(I:C) and LPS were used as stimulators for M1 macrophage polarization in the current study.

The concentration of LPS was determined according to our previous work (500 ng/ml) (37). A concentration gradient of Poly(I:C) was used to explore the optimum concentration of Poly(I:C) to induce M1- macrophage polarization. Western blot analysis indicated that 50 ng/ml was the optimum concentration for Poly(I:C)-derived M1 macrophages (**Figure S2**).

### Knockdown of IFIH1 Expression

We initially designed three different sequences specifically targeting mouse *IFIH1* according to GeneChem Co., Ltd. (http://www.genechem.com.cn; **Table S1**). The sequences of the shRNAs are indicated in **Table S2**. In brief, constructs expressing the shRNAs targeting endogenous *IFIH1* were encoded separately into the lentiviral vector GV248 (pLV3ltr-GFP-Puro-U6-Linear), which also encoded puromycin and green fluorescent protein (*GFP*). To check whether the targeted sequences were encoded in shRNAs, the lentiviral vectors were evaluated through sequencing.

RAW264.7 cells and BMDMs were transfected with lentivirus supernatant (multiplicity of infection = 50). To select the optimal shRNA, we detected the efficiency of *IFIH1* knockdown by RT-PCR and western blot (measuring *IFIH1* mRNA and protein expression) 3 days after transfection. The sequence of the most efficient ShRNA-*IFIH1* was finally identified as GCAGAAGCTGAGAAACAATGA. The control sequence was TTCTCCGAACGTGTCACGTT.

### Overexpression of IFIH1

We first amplified the full-length coding sequence of *IFIH1* (NM_001164477.1, 2931 bp) from RAW264.7 and BMDM cRNA through PCR. The primers for *IFIH1* were as follows:

T7-NheI-*Ifih1*-Pf: CTATAGGGAGACCCAAGCTGGCTAGCcgccaccATGTCGATTGTCTGTTCTGCAG
BGH-XhoI-*Ifih1*-pR: GTTTAAACGGGCCCTCTAGACTCGAGCTAATCTTCATCACTATACAAGCAGTATTCTG

The PCR products were purified and cloned into the pCMV3-GFP-Puro vector and then sequenced. The *IFIH1* overexpression vector and an empty vector were transfected into separate RAW264.7 cells and BMDMs. To evaluate the knockdown and overexpression efficiencies of transfection, RT-PCR and western blotting were used to measure the mRNA and protein expression of *IFIH1*, respectively, in every group of transfected RAW264.7 cells and BMDMs.

### RT-PCR

Total RNA was extracted from lung tissue samples or cells with TRIzol. Prime ScriptTM Trimester Mix (Takara, Japan) was utilized for reverse transcription of RNA. SYBR Premix Ex TaqTM11 (Takara, Japan) and a Step One Plus RT-PCR system (Life Technologies, USA) were utilized to perform RT-PCR based on the manufacturer’s instructions. The results were normalized to those for β-actin, and quantification was performed with the 2^-ΔΔCt of each sample^/2^-ΔΔCt of Ctrl^ method. The primers are shown in **Table S3**.

### Western Blot Analysis

We extracted total protein with RIPA lysis buffer. Then, we measured the protein concentrations of the cell lysates with a BCA protein assay (Beyotime, China) and equalized them. The proteins were separated by 8%–12% SDS-PAGE, blotted onto PVDF membranes, and subsequently incubated with primary antibodies, including antibodies against iNOS, *IFIH1, IRF3*, p-*IRF3*, and β-*tubulin* (Cell Signaling Technology, USA, 1:1000), followed by incubation with HRP-labeled secondary antibodies. ECL detection kits (Beyotime, China) were used to detect proteins and visualize the results on autoradiography film. ImageJ software (win64) was utilized to quantify the western blot band intensities.

### Flow Cytometry

RAW264.7 cells and BMDMs were collected with a scraper, blocked with a blocking agent (Miltenyi Biotech, Germany) for 10 min, and then incubated with a PE-conjugated anti-mouse *CD86* (1:200) or FITC-conjugated anti-mouse *F4/80* (1:200) antibody based on the manufacturers’ instructions. *F4/80* was utilized to identify macrophages, and *CD86* was utilized as the marker of M1 macrophages. The expression of *CD86* was calculated from the fluorescence intensity. All data were collected by flow cytometry (ACEA NovoCyte, China) using Novo Express (ACEA NovoCyte, China) and calculated using FlowJo software version X (Tree Star, USA).

### ELISA

Supernatants were collected from each culture condition. In these supernatants, *IL-1β* and *CCL2* were detected using enzyme-linked immunosorbent assay (ELISA) based on the manufacturer’s instructions (R&D Systems, USA).

### Elaboration of the Mechanism

To gain insight into the mechanisms underlying the regulatory role of the crucial gene, gene set enrichment analysis (GSEA) was conducted with mRNA datasets of macrophages, with *FDR*<0.05 as the cut-off value. GSEA is an algorithm designed to assess the concerted behavior of functionally related genes forming a set between two well-defined groups of samples. Because it does not rely on a “gene list” of interest but on the entire ranking of genes, GSEA has been shown to provide greater sensitivity to find gene expression changes of small magnitude that operate coordinately in specific sets of functionally related genes. GSEA calculates separate enrichment scores (ES) for each pairing of a sample based on the GSEABase R package (41, 42). Each ES stands for the degree to which the genes in the specific gene set (mechanisms) are coordinately up or down expressed within a sample. The GSEA database and algorithm introduction are clearly indicated in https://www.gsea-msigdb.org/gsea/index.jsp.

GSEA predicted that *IFIH1* regulates M1 macrophage polarization *via* the RIG-I pathway. To confirm our bioinformatic prediction, phosphorylated and nucleoprotein western blotting were implemented to investigate the crucial characteristics of RIG-I pathway activation (*IRF3* phosphorylation and *IRF3* translocation into the nucleus) on macrophages, transfected macrophages and controls.

To investigate whether LPS could also activate *IRF3 via IFIH1*, similar to Poly(I:C), these two stimulation agents were both utilized to construct M1-polarized macrophage models simultaneously. In addition, ST 2825 (a specific MyD88 dimerization inhibitor) was utilized to further explore whether LPS is involved in MyD88-dependent or independent mechanism activation of *IRF3*.

### Statistical Software

R x64 3.6.1 was used to process and analyze data and plot diagrams.

## Results

### Thirty-Six Candidate Genes May Be Associated With the Severity of ARDS and M1-Polarized Macrophages

**Figure 1:**
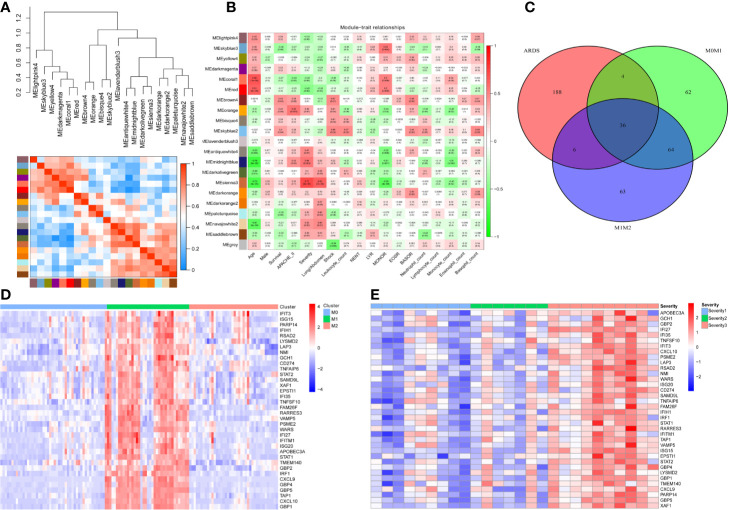
Thirty-six genes were associated with the severity of ARDS and M1-polarized macrophages. **(A)** All gene expression profiles from the 26 ARDS were classified as 20 phenotypes (models) *via* the WGCNA algorithm. **(B)** The heatmap of WGCNA results indicates that the MEsienna3 model (including 234 genes) was significantly associated with the severity of ARDS (*P*= 6×10^5^, Spearman correlation coefficient = 0.71). The numbers in the plots represent the correlation coefficient and *P* value. **(C)** The Venn diagram illustrates the overlap between genes significantly associated with the severity of ARDS (234 genes in the MEsienna3 model) and those specifically expressed in M1 macrophages (2-fold difference, *FDR*<0.05, compared with M0 and M2 macrophages). Thirty-six genes in the overlap were identified as candidate genes since they were simultaneously associated with ARDS and involved in M1 macrophage polarization. **(D)** The heatmap shows the expression profiles of the 36 candidate genes in M0-, M1-, and M2-polarized macrophages derived from 125 volunteers’ alveolar macrophages. The expression profiles of each gene were then multiplied by the corresponding log2 value to yield final gene expression values. **(E)** The heatmap shows the expression profiles of the 36 candidate genes in 26 patients with different severities of ARDS. The expression profiles of each gene were then multiplied by the corresponding log2 value to yield final gene expression values.

To screen genes associated with the severity of ARDS, we utilized the weighted gene coexpression network analysis (WGCNA) algorithm on all gene expression profiles from 26 ARDS patients. The cluster heatmap in **Figure 1A** illuminated that all gene expression profiles from the 26 ARDS were classified as 20 phenotypes (models) *via* the WGCNA algorithm. A correlation heatmap of the WGCNA results indicated that the MEsienna3 model (including 234 genes) was significantly associated with the severity of ARDS (*P*= 6×10^5^, Spearman correlation coefficient = 0.71), as shown in **Figure 1B**. These 26 ARDS patients comprised nine mild ARDS patients, seven moderate ARDS patients and 10 severe ARDS patients graded by the Berlin definition of ARDS. The detailed demographic and clinical characteristics of the study sample population grouped according to ARDS diagnosis are listed in **Table S4**.

To investigate the genes potentially involved in M1 macrophage polarization, we identified significantly differentially expressed genes between M1 macrophages and M0 and M2 macrophages [2-fold difference, false discovery rate (*FDR*<0.05)]. After the search strategy and inclusion criteria were executed, one mRNA dataset of human alveolar macrophages, one mRNA dataset of human microglia, nine mRNA datasets of human peripheral blood monocyte-derived macrophages and two mRNA datasets of mouse bone marrow-derived macrophages were screened (**Table S5**). The GSE46903 dataset was enrolled as the macrophage training dataset since this dataset had the largest sample size (human alveolar macrophages from 125 volunteers). Simultaneously, other datasets were utilized as macrophage validation datasets. One hundred M1-distinct genes were identified *via* differential expression gene analysis of the macrophage training dataset (human alveolar macrophages from 125 volunteers).

Furthermore, to find candidate genes simultaneously associated with ARDS and M1 macrophages, a Venn diagram was plotted to capture the overlap between 234 genes in the MEsienna3 model (significantly associated with the severity of ARDS) and 100 genes specifically expressed in M1 macrophages (**Figure 1C**). The Venn diagram identified 36 candidate genes simultaneously associated with ARDS and M1 macrophages. The expression profiles of the 36 candidate genes in different subsets of macrophages and different severities of ARDS are described in **Figures 1D, E**, respectively.

### IFIH1 Was Identified as the Crucial Candidate for Further Investigation

**Figure 2:**
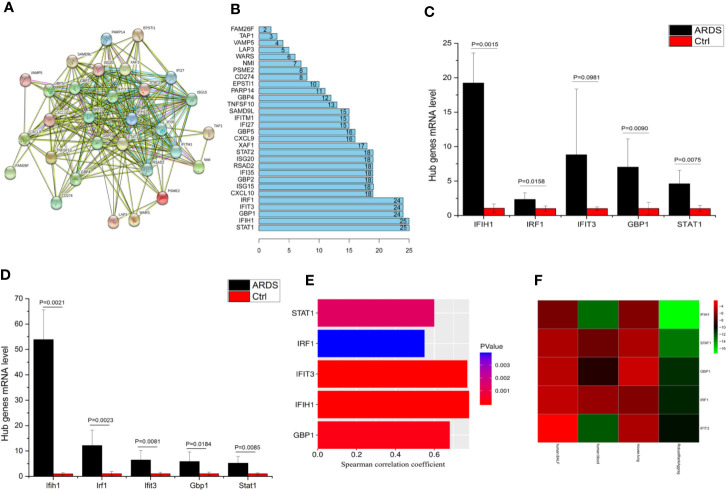
*IFIH1* was identified as a hub gene involved in ARDS. **(A)** The network plot represents interactions among 36 genes according to protein interaction analyses. **(B)** The histogram illustrates the hub genes. The numbers in the histogram represent the numbers of interactions among the 36 genes based on protein interaction analyses. There is an inflection point between *IRF1* and *CXCL10*. Therefore, the top 5 genes (*STAT1*, *IFIH1*, *GBP1*, *IFIT3*, and *IRF1*) were selected as the hub genes since they have the most interactions. **(C)** The mRNA levels of *IFIH1*, *IRF1*, *IFIT3*, *GBP1*, and *STAT1* in bronchoalveolar lavage fluid (BALF) from patients with ARDS (n=6) and patients without ARDS (postoperative patients, n=6) were measured by qRT-PCR. The mRNA expression was calculated based on 2^-ΔΔCt of each sample^/2^-ΔΔCt of Ctrl^. Student’s t test indicated that the mRNA levels of *IFIH1*, *GBP1*, and *STAT1* were obviously upregulated in the BALF of the ARDS patients (*P*<0.05). All error bars represent the SDs. **(D)** The mRNA levels of *Ifih1*, *Irf1*, *Ifit3*, *Gbp1* and *Stat1* in lung tissue homogenates from ARDS model mice (n=6) and control mice (n=6) were measured by qRT-PCR. Student’s t test indicated that the mRNA levels of *Ifih1*, *Irf1*, *Ifit3*, *Gbp1*, and *Stat1* were obviously upregulated in the ARDS mice (*P* < 0.05). All error bars represent the SDs. **(E)** The plots show the associations of *IFIH1*, *IRF1*, *IFIT3*, *GBP1*, and *STAT1* mRNA expression profiles in 26 ARDS patients’ peripheral blood with the severity of ARDS. Plot length represents the correlation coefficient between the mRNA expression profiles of each gene and the severity of ARDS. Plot color depth represents the *P* value from Spearman’s correlation analysis of the expression profiles of each gene and the severity of ARDS. **(F)** The heatmap represents the *P* values from panels **(C–E)** and the interrelated *P* values. The RobustRankAggreg algorithm helped us find the gene with the strongest ARDS-related correlation (minimum *P* value and maximum Spearman correlation coefficient). The interrelated *P* value was calculated *via* the RobustRankAggreg algorithm based on the *P* values from human and animal experiments.

To determine these crucial genes (hub genes) among the 36 candidates, we plotted a PPI network diagram *via* STRING, as shown in **Figure 2A**. Then, the total connectivity degree of each node in the network was calculated by connectivity degree analysis (number of neighbors), as shown in **Figure 2B**. There was an inflection point between *IRF1* and *CXCL10*, which indicated that the genes above this inflection point had a markedly higher degree of connectivity (more number of interactions) than the genes below (**Figure 2B**). Therefore, *STAT1, IFIH1, GBP1, IFIT3*, and *IRF1* were identified as hub genes.

To further validate the expression distribution of five hub genes in M0/M1/M2 macrophages, 11 external mRNA datasets (nine human macrophage and two mouse macrophage datasets) were defined as macrophage validation datasets. The results revealed that the expression of the five hub genes was markedly upregulated in M1-polarized macrophages (*FDR*<0.05), as shown in **Figure S3**.

To validate whether the hub genes are involved in ARDS, the mRNA levels of *IFIH1, IRF1, IFIT3, GBP1*, and *STAT1* in BALF from patients with ARDS (n=6) and mechanically ventilated patients without ARDS (postoperative patients, n=6) were measured by real-time quantitative PCR (qRT-PCR). The detailed information of ARDS and control patients were shown in **Table S6**. A t test indicated that the levels of *IFIH1, IRF1, GBP1*, and *STAT1* were significantly upregulated in the ARDS samples (*P*<0.05), as shown in **Figure 2C**. Additionally, animal experiments indicated that the mRNA levels of *Ifih1, Irf1, Ifit3, Gbp1, and Stat1* were obviously upregulated in lung tissue homogenates from the ARDS model mice (n=6) compared with those from control mice (n=6), as shown in **Figure 2D**. In addition, **Figure 2E** shows the associations of the *IFIH1, IRF1, IFIT3, GBP1*, and *STAT1* mRNA expression profiles with the severity of ARDS.

To identify the gene with the strongest ARDS-related correlation, we utilized the RobustRankAggreg algorithm to calculate interrelated *P* values based on the *P* values from the human and animal experiments. Then, the five hub genes were ranked according to interrelated *P* values from minimum to maximum, as shown in **Figure 2F**. *IFIH1* was selected because it had the minimum interrelated *P* value and maximum Spearman correlation coefficient. Additionally, immunofluorescence indicated that *IFIH1* protein expression was significantly upregulated in M1-polarized macrophages, as shown in **Figure S4**.

### IFIH1 Contributes to Poly(I:C)-Induced and LPS-Induced M1 Macrophage Polarization

To validate whether *IFIH1* could affect M1 macrophage polarization, shRNA) sequences and plasmid expression vectors for *IFIH1* were introduced to construct transfected RAW264.7 cell lines and BMDMs with *IFIH1* overexpression or knockdown, respectively, and these cell lines were treated with LPS induction for 24 h. As shown in **Figure S5–S8**, the shRNA sequence and plasmid expression vectors were able to silence and overexpress *IFIH1*, respectively, in the RAW264.7 cell line and BMDMs.

**Figure 3:**
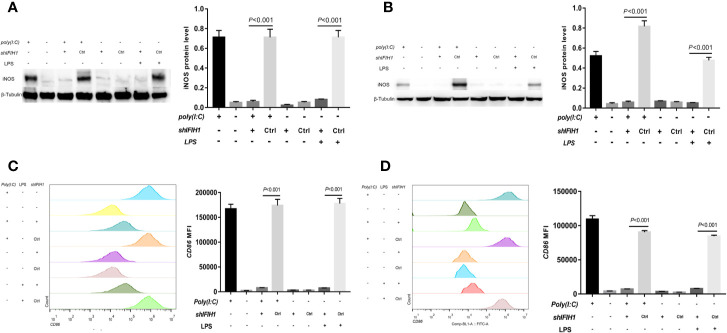
Knockdown of *IFIH1* expression attenuates both Poly(I:C)-induced and LPS-induced M1 macrophage polarization. To evaluate whether knockdown of *IFIH1* expression attenuates M1 macrophage polarization, we constructed RAW264.7 cell lines stably transfected with shRNAs specifically targeting *IFIH1* or control shRNAs to create knockdown and control cell lines, respectively. Moreover, bone marrow–derived macrophages (BMDMs) were transfected with shRNAs specifically targeting *IFIH1* or control shRNAs to create *IFIH1* knockdown and control primary macrophages, respectively. Then, each group of cell lines was stimulated with Poly(I:C) or LPS for 24 h. **(A)** Western blotting was used to detect the expression of *iNOS* in RAW264.7 cells, a stably transfected RAW264.7 cell line with *IFIH1* knockdown, and a control shRNA cell line. The quantitative analysis of western blotting indicated that both Poly(I:C)-induced and LPS-induced *iNOS* expression in RAW264.7 cells was markedly decreased after *IFIH1* silencing (*P*<0.05). In addition, the vector transfection of sh*IFIH1* and control shRNA did not influence *iNOS* expression in RAW264.7 cells. **(B)** Western blotting was used to detect the expression of *iNOS* in BMDMs, transfected BMDMs with *IFIH1* knockdown, and control shRNA BMDMs. The quantitative analysis of western blotting indicated that both Poly(I:C)-induced and LPS-induced *iNOS* expression in BMDMs was markedly decreased after *IFIH1* silencing (*P*<0.05). In addition, the vector transfection of sh*IFIH1* and control shRNA did not influence *iNOS* expression in BMDMs. **(C)** Flow cytometric analysis was performed to analyze the MFI of *CD86* on *F4/80*+ RAW264.7 cells. Quantitative analysis indicated that *CD86* expression in both Poly(I:C)-induced and LPS-induced M1-polarized RAW264.7 cell models was markedly decreased after *IFIH1* silencing (*P*<0.05). In addition, the vector transfection of sh*IFIH1* and control shRNA did not influence *CD86* expression in RAW264.7 cells. **(D)** Flow cytometric analysis was performed to analyze the MFI of *CD86* on *F4/80*+ BMDMs. Quantitative analysis indicated that *CD86* expression in both Poly(I:C)-induced and LPS-induced M1-polarized BMDM models was markedly decreased after *IFIH1* silencing (*P*<0.05). In addition, the vector transfection of sh*IFIH1* and control shRNA did not influence *CD86* expression in BMDMs.

Western blot analysis indicated that both Poly(I:C)-induced and LPS-induced *iNOS* expression in RAW264.7 cells and BMDMs was markedly decreased after silencing *IFIH1* compared with control treatment (control shRNA transfection; *P*<0.05, **Figures 3A, B**). In addition, the vector transfection of sh*IFIH1* and control shRNA did not influence *iNOS* expression in RAW264.7 cells and BMDMs.

**Figure 4:**
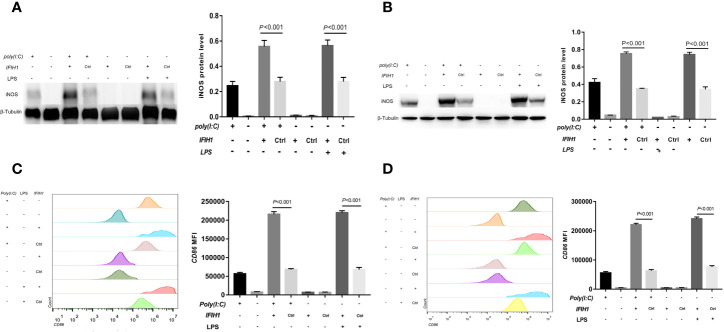
Overexpression of *IFIH1* promotes both Poly(I:C)-induced and LPS-induced M1 macrophage polarization. To investigate whether overexpression of *IFIH1* could promote M1 macrophage polarization, we constructed transfected RAW264.7 cell lines and bone marrow–derived macrophages (BMDMs) with plasmid *IFIH1* expression vectors and control blank vectors. Then, each group of cells was stimulated with Poly(I:C) or LPS for 24 h for 24 h. **(A)** Western blotting was performed to detect the expression of *iNOS* in RAW264.7 cells, a stably transfected RAW264.7 cell line overexpressing *IFIH1*, and a control cell line. The quantitative analysis indicated that both Poly(I:C)-induced and LPS-induced *iNOS* expression in RAW264.7 cells was significantly increased after overexpression of *IFIH1* (*P*<0.05). In addition, only *IFIH1* expression without Poly(I:C) or LPS stimulation did not influence *iNOS* expression in the RAW264.7 cell line. **(B)** Western blotting was performed to detect the expression of *iNOS* in BMDMs, transfected BMDMs overexpressing *IFIH1*, and control BMDMs. The quantitative analysis indicated that both Poly(I:C)-induced and LPS-induced *iNOS* expression in BMDMs was significantly increased after overexpression of *IFIH1* (*P*<0.05). In addition, only *IFIH1* expression without Poly(I:C) or LPS stimulation could not influence *iNOS* expression in BMDMs. **(C)** Flow cytometric analysis was performed to analyse the MFI of *CD86* on *F4/80*+ RAW264.7 cells. Quantitative analysis indicated that *CD86* expression in both Poly(I:C)-induced and LPS-induced M1-polarized RAW264.7 cell models was markedly increased after overexpression of *IFIH1* (*P*<0.05). In addition, only *IFIH1* expression without Poly(I:C) or LPS stimulation did not influence *CD86* expression in RAW264.7 cells. **(D)** Flow cytometric analysis was performed to analyse the MFI of *CD86* on *F4/80*+ BMDMs. Quantitative analysis indicated that *CD86* expression in both Poly(I:C)-induced and LPS-induced M1-polarized BMDMs models was markedly increased after overexpression of *IFIH1* (*P* < 0.05). In addition, only *IFIH1* expression without Poly(I:C) or LPS stimulation could not influence *CD86* expression in BMDMs. Statistical data are from three independent experiments, and the bar indicates the SD.

Similarly, compared with that measured following control treatment (transfection with blank vectors), both Poly(I:C)-induced and LPS-induced *iNOS* expression measured in RAW264.7 cells and BMDMs after overexpression of *IFIH1* was significantly increased (*P*<0.05, **Figures 4A, B**). In addition, only *IFIH1* expression without Poly(I:C) or LPS stimulation could not influence *iNOS* expression in the RAW264.7 cell line and BMDMs.

To confirm our findings, we examined other M1 phenotypic markers. Flow cytometry results illustrated that the expression of *CD86* in both Poly(I:C)-induced and LPS-induced M1-polarized cell models was markedly decreased after silencing *IFIH1* in the RAW264.7 cell line and BMDMs (*P*<0.05, **Figures 3C, D**).

In contrast, CD86 expression was markedly increased in both Poly(I:C)-induced and LPS-induced M1-polarized cell models after overexpression of *IFIH1* in the RAW264.7 cell line and BMDMs (*P*<0.05, **Figures 4C, D**). Additionally, only *IFIH1* expression without Poly(I:C) or LPS stimulation could not influence *CD86* expression in the RAW264.7 cell line and BMDMs.

The above results revealed that *IFIH1* is the regulatory molecule involved in Poly(I:C)-induced and LPS-induced macrophage polarization from M0 to M1.

### IFIH1 Regulates Inflammatory Cytokine Secretion From Macrophages

To further assess the pro-inflammatory role of *IFIH1* in macrophages, ELISAs were performed to detect the interleukin (IL)-1β protein (an inflammatory cytokine secreted by M1 macrophages) and C-C motif chemokine ligand 2 (*CCL2*, a chemokine secreted by M1 macrophages) in culture medium from macrophages, transfected macrophages and controls.

**Figure 5:**
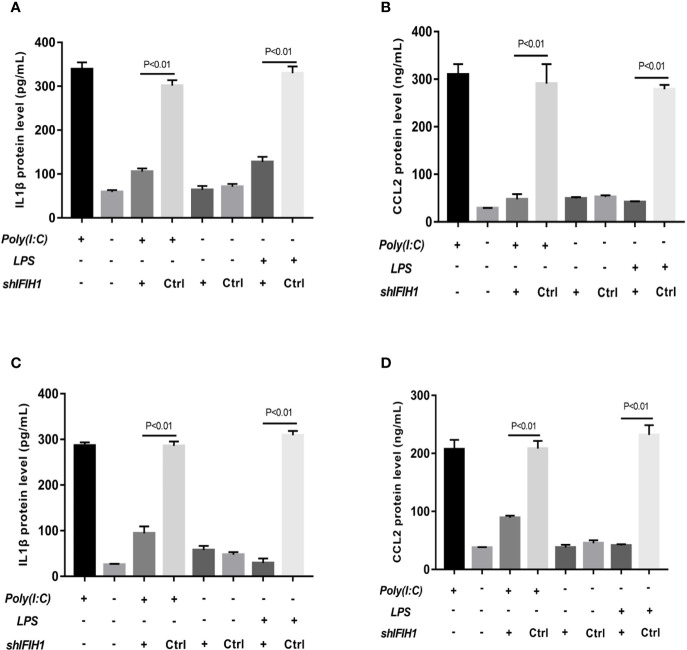
Knockdown of *IFIH1* expression attenuates inflammatory cytokine secretion from macrophages. ELISA detected the expression of *IL-1β* and *CCL2* in culture medium from RAW264.7 cells, a stably transfected RAW264.7 cell line with *IFIH1* knockdown, and a control shRNA cell line. Meanwhile, *IL-1β* and *CCL2* were also measured in culture medium from bone marrow–derived macrophages (BMDMs) transfected with sh*IFIH1* BMDMs and control shRNA control. **(A)** The ELISA results revealed that the protein expression of *IL-1β* (an inflammatory cytokine secreted by M1 macrophages) was markedly decreased in both Poly(I:C)-induced and LPS-induced M1-polarized RAW264.7 cell models after *IFIH1* silencing (*P*<0.05). In addition, the vector transfection of sh*IFIH1* and control shRNA did not influence IL-1β expression in the RAW264.7 cell line. **(B)** ELISA results revealed that the protein expression of *CCL2* (a chemokine secreted by M1 macrophages) was markedly decreased in both Poly(I:C)-induced and LPS-induced M1-polarized RAW264.7 cell models after *IFIH1* silencing (*P*<0.05). In addition, the vector transfection of sh*IFIH1* and control shRNA did not influence *CCL2* expression in the RAW264.7 cell line. **(C)** The ELISA results revealed that the protein expression of *IL-1β* (an inflammatory cytokine secreted by M1 macrophages) was markedly decreased in both Poly(I:C)-induced and LPS-induced M1-polarized BMDMs models after *IFIH1* silencing (*P*<0.05). In addition, the vector transfection of sh*IFIH1* and control shRNA did not influence IL-1β expression in BMDMs. **(D)** ELISA results revealed that the protein expression of *CCL2* (a chemokine secreted by M1 macrophages) was markedly decreased in both Poly(I:C)-induced and LPS-induced M1-polarized BMDMs models after *IFIH1* silencing (*P*<0.05). Statistical data are from three independent experiments, and the bar indicates the SD. In addition, the vector transfection of sh*IFIH1* and control shRNA did not influence *CCL2* expression in BMDMs.

**Figure 6:**
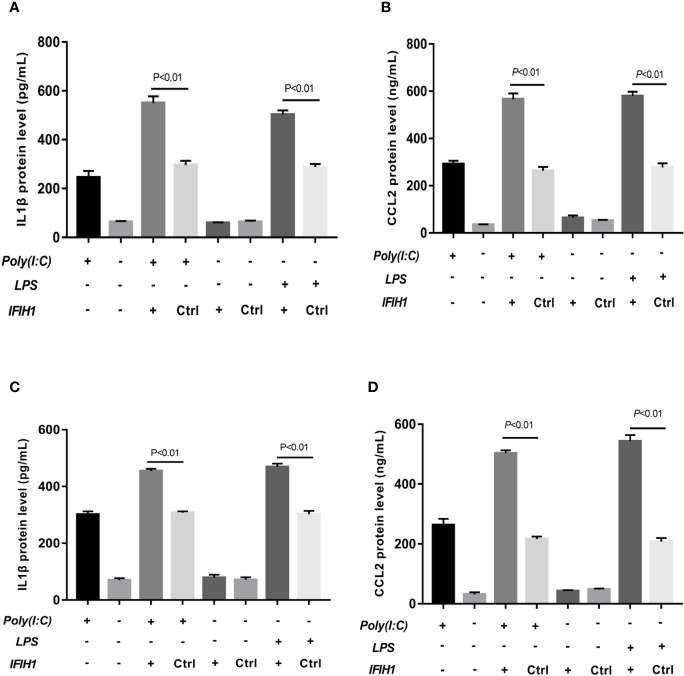
Overexpression of *IFIH1* promotes inflammatory cytokine secretion from macrophages. ELISA detected the expression of *IL-1β* and *CCL2* in culture medium from RAW264.7 cells, a transfected RAW264.7 cell line with *IFIH1* expression vectors, and a control blank vector cell line. Meanwhile, *IL-1β* and *CCL2* were also measured in culture medium from bone marrow–derived macrophages (BMDMs) transfected with *IFIH1* expression vectors and control blank vectors. **(A)** The ELISA results revealed that the protein expression of *IL-1β* (an inflammatory cytokine secreted by M1 macrophages) was markedly increased in both Poly(I:C)-induced and LPS-induced M1-polarized RAW264.7 cell models after *IFIH1* overexpression of *IFIH1* (*P*<0.05). In addition, only *IFIH1* expression without Poly(I:C) or LPS stimulation did not influence *IL-1β* expression in RAW264.7 cells. **(B)** ELISA results revealed that the protein expression of *CCL2* (a chemokine secreted by M1 macrophages) was markedly increased in M1-polarized RAW264.7 cell models after *IFIH1* overexpression of IFIH1 (*P*<0.05). In addition, only *IFIH1* expression without Poly(I:C) or LPS stimulation could not influence *CCL2* expression in RAW264.7 cells. **(C)** The ELISA results revealed that the protein expression of *IL-1β* (an inflammatory cytokine secreted by M1 macrophages) was markedly increased in both Poly(I:C)-induced and LPS-induced M1-polarized BMDMs models after *IFIH1* overexpression of *IFIH1* (*P*<0.05). In addition, only *IFIH1* expression without Poly(I:C) or LPS stimulation did not influence *IL-1β* expression in BMDMs. **(D)** ELISA results revealed that the protein expression of *CCL2* (a chemokine secreted by M1 macrophages) was markedly increased in both Poly(I:C)-induced and LPS-induced M1-polarized BMDMs models after overexpression of *IFIH1* (*P*<0.05). In addition, only *IFIH1* expression without Poly(I:C) or LPS stimulation could not influence *CCL2* expression in BMDMs. Statistical data are from three independent experiments, and the bar indicates the SD.

The results revealed that the expression of *IL-1β* and *CCL2* was markedly decreased in both Poly(I:C)-induced and LPS-induced M1-polarized cell models after silencing *IFIH1* in the RAW264.7 cell line and BMDMs (*P*<0.05, **Figure 5**). Similar to the above results, the expression of *IL-1β* and *CCL2* was significantly increased in both Poly(I:C)-induced and LPS-induced M1-polarized cell models after overexpression of *IFIH1* in the RAW264.7 cell line and BMDMs (*P*<0.05, **Figure 6**). Additionally, only *IFIH1* expression without Poly(I:C) or LPS stimulation could not influence *IL-1β* and *CCL2* expression in the RAW264.7 cell line and BMDMs.

Collectively, these data indicate that *IFIH1* regulates the inflammatory function of macrophages.

### IFIH1 Regulates Macrophage Polarization by Activating IRF3

To gain insight into the mechanisms underlying the regulatory role of *IFIH1*, we conducted GSEA of the mRNA dataset of human macrophages (GSE46903). The GSEA results indicated that the *RIG-I* pathway was significantly activated and that the genes in the *RIG-I* pathway were obviously enriched when *IFIH1* expression was upregulated (*FDR*<0.05, **Figure S9**).

It is well known that the characteristics of *RIG-I* pathway activation include *IRF3* phosphorylation and *IRF3* translocation into the nucleus. Therefore, phosphorylated and nucleoprotein western blotting was performed to validate whether IFIH1 could affect Poly(I:C)-induced or LPS-induced *IRF3* activation.

**Figure 7:**
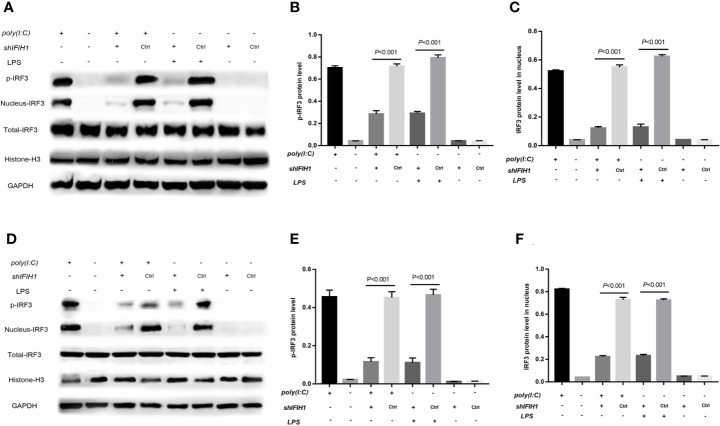
Knockdown of *IFIH1* suppressed *IRF3* phosphorylation and *IRF3* translocation into the nucleus in response to both Poly(I:C)-induced and LPS-induced conditions. GSEA suggested that *IFIH1* regulated M1 macrophage polarization *via* the RIG-I pathway. Previous studies have demonstrated that the indicators of RIG-I pathway activation include *IRF3* phosphorylation and *IRF3* translocation into the nucleus. Therefore, phosphorylated and nucleoprotein western blotting was performed to validate whether knockdown of *IFIH1* could suppress Poly(I:C)-induced or LPS-induced *IRF3* activation. Histone H3 and *GAPDH* were used as nuclear and total reference proteins, respectively. **(A)** Western blotting was performed to detect the levels of phosphorylated IRF3 and the expression of *IRF3* in the nucleus and whole *IRF3* in each group of RAW264.7 cells. **(B)** Quantitative analysis of phosphorylated *IRF3* indicated that both Poly(I:C)-induced and LPS-induced phosphorylation of *IRF3* in RAW264.7 cells were markedly decreased after *IFIH1* silencing compared with control treatment (*P*<0.05). In addition, the vector transfection of sh*IFIH1* and control shRNA did not influence the phosphorylation of *IRF3* in RAW264.7 cells. **(C)** Quantitative analysis of *IRF3* in the cell nucleus indicated that both Poly(I:C)-induced and LPS-induced expression of *IRF3* in RAW264.7 cell nuclei were markedly decreased after *IFIH1* silencing compared with control treatment (*P*<0.05). In addition, the vector transfection of sh*IFIH1* and control shRNA did not influence the expression of *IRF3* in the RAW264.7 cell nucleus. **(D)** Western blotting was performed to detect the levels of phosphorylated *IRF3* and the expression of *IRF3* in the nucleus and whole *IRF3* in each group of bone marrow–derived macrophages (BMDMs). **(E)** Quantitative analysis of phosphorylated *IRF3* indicated that both Poly(I:C)-induced and LPS-induced phosphorylation of *IRF3* in BMDMs were markedly decreased after *IFIH1* silencing compared with control treatment (*P*<0.05). In addition, the vector transfection of sh*IFIH1* and control shRNA did not influence the phosphorylation of *IRF3* in BMDMs. **(F)** Quantitative analysis of *IRF3* in the cell nucleus indicated that both Poly(I:C)-induced and LPS-induced expression of *IRF3* in BMDM nuclei were markedly decreased after *IFIH1* silencing compared with control treatment (*P*<0.05). In addition, the vector transfection of sh*IFIH1* and control shRNA did not influence the expression of *IRF3* in BMDM nuclei. Statistical data are from three independent experiments, and the bar indicates the SD.

Compared with the control group, the silenced group showed markedly decreased Poly(I:C)-induced and LPS-induced phosphorylation of *IRF3* in RAW264.7 cells and BMDMs by western blot analysis (*P*<0.05, **Figure 7**). Additionally, compared with control treatments, Poly(I:C)–induced and LPS-induced *IRF3* expression in the RAW264.7 and BMDMs cell nuclei were significantly downregulated after silencing *IFIH1* (*P*<0.05, **Figure 7**).

**Figure 8:**
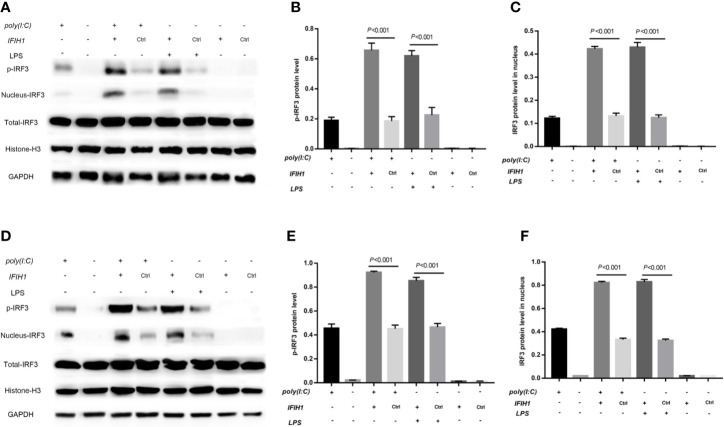
Overexpression of *IFIH1* promotes *IRF3* phosphorylation and *IRF3* translocation into the nucleus in response to both Poly(I:C)-induced and LPS-induced conditions. Furthermore, phosphorylated and nucleoprotein western blotting was performed to validate whether overexpression of *IFIH1* could promote Poly(I:C)-induced or LPS-induced *IRF3* activation. **(A)** Western blotting was performed to detect the levels of phosphorylated *IRF3* and the expression of *IRF3* in the nucleus and whole *IRF3* in each group of RAW264.7 cells. **(B)** Quantitative analysis of phosphorylated *IRF3* indicated that both Poly(I:C)-induced and LPS-induced phosphorylation of *IRF3* in RAW264.7 cells were markedly increased after overexpression of *IFIH1* compared with control treatment (*P*<0.05). In addition, only *IFIH1* expression without Poly(I:C) or LPS stimulation could not influence the phosphorylation of *IRF3* in RAW264.7 cells. **(C)** Quantitative analysis of *IRF3* in the cell nucleus indicated that both Poly(I:C)-induced and LPS-induced expression of *IRF3* in RAW264.7 cell nuclei were markedly increased after overexpression of *IFIH1* compared with control treatment (*P*<0.05). In addition, only *IFIH1* expression without Poly(I:C) or LPS stimulation could not influence the expression of *IRF3* in the RAW264.7 nucleus. **(D)** Western blotting was performed to detect the levels of phosphorylated *IRF3* and the expression of *IRF3* in the nucleus and whole *IRF3* in each group of bone marrow–derived macrophages (BMDMs). **(E)** Quantitative analysis of phosphorylated *IRF3* indicated that both Poly(I:C)-induced and LPS-induced phosphorylation of *IRF3* in BMDMs were markedly increased after overexpression of *IFIH1* compared with control treatment (*P*<0.05). In addition, only *IFIH1* expression without Poly(I:C) or LPS stimulation could not influence the phosphorylation of *IRF3* in BMDMs. **(F)** Quantitative analysis of *IRF3* in the cell nucleus indicated that both Poly(I:C)-induced and LPS-induced expression of *IRF3* in BMDM nuclei were markedly increased after overexpression of *IFIH1* compared with control treatment (*P*<0.05). In addition, only *IFIH1* expression without Poly(I:C) or LPS stimulation did not influence the expression of *IRF3* in BMDM nuclei. Statistical data are from three independent experiments, and the bar indicates the SD.

In contrast, phosphorylation of *IRF3* and *IRF3* expression in the cell nucleus were markedly increased in both Poly(I:C)-induced and LPS-induced M1-polarized cell models after overexpression of *IFIH1* in the RAW264.7 cell line and BMDMs (*P*<0.05, **Figure 8**). Additionally, only *IFIH1* expression without Poly(I:C) or LPS stimulation could not influence the activation of *IRF3* in the RAW264.7 cell line and BMDMs.

These results suggest that *IFIH1* regulates macrophage polarization by activating *IRF3*.

### LPS-Induced Activation of IRF3 Depends on the MyD88 Pathway

Previous studies have robustly demonstrated that *IFIH1* activation of *IRF3* depends on dsRNA or RNA mimics. The current study first found that LPS could also activate *IRF3 via IFIH1*. It is well known that the classical pathway of LPS stimulation is MyD88-dependent. Therefore, ST 2825 (a specific MyD88 dimerization inhibitor) was utilized to further explore whether LPS is involved in MyD88-dependent or independent mechanism activation of *IRF3*.

**Figure 9:**
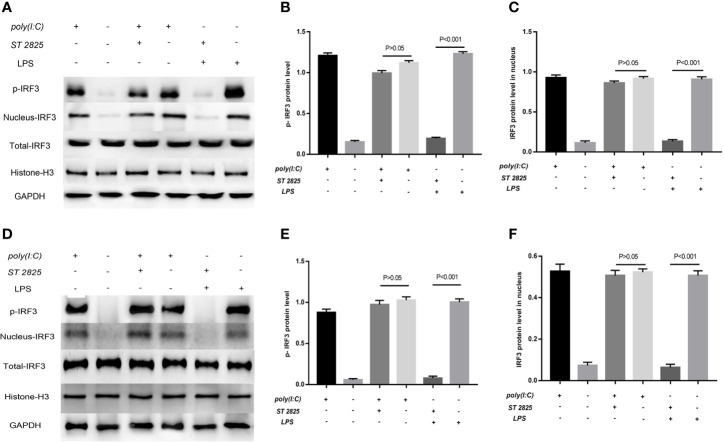
LPS-induced activation of *IRF3* depends on the MyD88 pathway. Previous studies robustly demonstrated that *IFIH1* activation of *IRF3* depends on dsRNA or RNA mimics. The current study first found that LPS could also activate *IRF3 via IFIH1*. It is well known that the classical pathway of LPS stimulation is MyD88-dependent. Therefore, ST 2825 (a specific MyD88 dimerization inhibitor) was utilized to further explore whether LPS is involved in MyD88-dependent or independent mechanism activation of *IRF3*. **(A)** Western blotting was performed to detect the levels of phosphorylated *IRF3* and the expression of *IRF3* in the nucleus and whole *IRF3* in each group of RAW264.7 cells. **(B)** Quantitative analysis of phosphorylated *IRF3* indicated that blocking the MyD88 pathway did not affect the Poly(I:C)-induced phosphorylation of *IRF3*, but the MyD88 inhibitor significantly decreased the LPS-induced phosphorylation of *IRF3* compared with the control treatment in RAW264.7 cells (*P*<0.05). **(C)** Quantitative analysis of *IRF3* in the cell nucleus indicated that blocking the MyD88 pathway did not affect the Poly(I:C)-induced expression of *IRF3* in the RAW264.7 cell nucleus, but the MyD88 inhibitor significantly decreased the expression of *IRF3* in the RAW264.7 cell nucleus compared with the control treatment (*P*<0.05). **(D)** Western blotting was performed to detect the levels of phosphorylated *IRF3* and the expression of *IRF3* in the nucleus and whole *IRF3* in each group of bone marrow–derived macrophages (BMDMs). **(E)** Quantitative analysis of phosphorylated *IRF3* indicated that blocking the MyD88 pathway did not affect the Poly(I:C)-induced phosphorylation of *IRF3*, but the MyD88 inhibitor significantly decreased the LPS-induced phosphorylation of *IRF3* compared with the control treatment in BMDMs (*P*<0.05). **(F)** Quantitative analysis of *IRF3* in the cell nucleus indicated that blocking the MyD88 pathway did not affect the Poly(I:C)-induced expression of *IRF3* in the BMDM nucleus, but the MyD88 inhibitor significantly decreased the expression of *IRF3* in the BMDM nucleus compared with the control treatment (*P*<0.05). Statistical data are from three independent experiments, and the bar indicates the SD.

Western blot analysis of phosphorylated *IRF3* indicated that blocking the MyD88 pathway did not affect the Poly(I:C)-induced phosphorylation of *IRF3*, but the MyD88 inhibitor significantly decreased the LPS-induced phosphorylation of *IRF3* compared with the control treatment in RAW264.7 cells and BMDMs (*P*<0.05) (**Figure 9**).

Similarly, the quantitative analysis of nuclear western blot indicated that blocking the MyD88 pathway did not affect the Poly(I:C)-induced expression of *IRF3* in the RAW264.7 cell and BMDMs nucleus, but the MyD88 inhibitor significantly decreased the expression of *IRF3* in RAW264.7 cells and BMDMs compared with the control treatment (*P*<0.05). (**Figure 9**).

These results indicates that LPS-induced activation of *IFIH1-IRF3* depends on MyD88 pathway, and Poly(I:C)-induced activation of *IFIH1-IRF3* is the independent mechanism.

## Discussion

ARDS is considered a dysregulated systemic inflammatory host response to infections or other reasons for induced lung injury. Indeed, a switch from the M0 macrophage phenotype towards the M1 macrophage phenotype plays a crucial role in the initial stage of the inflammatory process of ARDS. Therefore, we conducted interrelated bioinformatic analysis to find that *IFIH1* is simultaneously associated with ARDS and M1 macrophages. Further experiments on RAW264.7 cells and BMDMs confirmed that *IFIH1* is a novel regulator for promoting M1 macrophage polarization *via IRF3* activation. In addition, previous studies have demonstrated that activation of *IFIH1*-*IRF3* is stimulated by viral RNAs or RNA mimics (31–35). Surprisingly, the current study finds that LPS can also induce *IFIH1*-*IRF3* activation *via a* MyD88-dependent mechanism. These findings are validated on two types of macrophages, RAW264.7 cells and BMDMs, which expand the knowledge on the inflammatory roles of *IFIH1* and *IRF3*.

*IFIH1* is a cytosolic receptor responsible for binding viral RNA and activating *IFN* regulatory factor 3 (*IRF3*) and NF-κB, resulting in the induction of inflammatory and antiviral genes. In contrast to previous studies (RNA mimic-induced *IFIH1*-*IRF3* activation) (42, 43), here, we demonstrate that LPS can also trigger *IRF3* activation *via IFIH1*. Interestingly, LPS and RNA mimic stimulate distinct signaling pathways, one leading to MyD88-dependent *IRF3* activation and the other to induction of independent *IRF3* activation. The MyD88 pathway has been confirmed as the classical downstream mechanism in response to LPS activation. Future investigations could focus on molecular production of the MyD88 pathway downstream for exploration of novel IFIH1-binding molecules.

Previous studies indicated that *IFIH1* was an “adaptor” in host defense involved in identifying a pathogen or tissue damage, further inducing inflammation (31–33). As accumulative evidence for the pro-inflammatory function of *IFIH1*, the current study further uncovers *IFIH1* as a novel molecular regulator in the development of macrophage polarization from M0 to M1. Interestingly, we found that only *IFIH1* expression without LPS or RNA mimic stimulation did not affect *IRF3* activation or M1 macrophage polarization. This result indicates that *IFIH1* is a signal transducing molecule rather than an activator in M1 macrophage polarization, the RIG-I pathway and inflammatory networks.

In addition to the detection of viral RNA, *IFIH1* has been demonstrated to be involved in some inflammatory diseases, such as periodontitis and systemic lupus erythematosus (32, 33, 43, 44). Our results show that *IFIH1* expression is markedly associated with ARDS severity, which increases the evidence of a link between *IFIH1* and inflammatory diseases. Additionally, *IFIH1* could be an inflammatory biomarker to represent the immune situation of ARDS, which is strongly consistent with the biological function of *IFIH1* (M1 macrophage polarization). This result provides an opportunity to develop bedside molecular tests for further ARDS monitoring.

Several limitations exist in the current study. On the one hand, this study lacked *IFIH1* knockout mice to further investigate the function of *IFIH1* and its associated biological mechanism *in vivo*. In addition, other molecular mechanisms underlying the regulatory role of *IFIH1* could be explored *in vitro*. In addition, detailed activators of *IFIH1* downstream of the MyD88 pathway have not been found.

## Conclusions

In summary, we identified *IFIH1* as a novel regulator of M1 polarization that acts by modulating *IRF3* activation in RAW264.7 cells and BMDMs. Moreover, LPS and RNA mimics trigger IFIH1-IRF3 *via* distinct signaling pathways, one leading to the MyD88-dependent pathway and the other leading to the induction of an independent mechanism. Additionally, *IFIH1* was found to have a strong relevance to ARDS severity. These findings could lead to intervention targets, with further experiments being warranted to assess the translational value of this work.

## Data Availability Statement

The severity of ARDS and mRNA data matrix could be downloaded in supplemental data: https://www.frontiersin.org/articles/10.3389/fimmu.2020.580838/full#supplementary-material

The high-throughput experiments of macrophage polarization in this study could be found in the following links:

GSE46903 https://www.ncbi.nlm.nih.gov/geo/query/acc.cgi?acc=GSE46903

GSE76737 https://www.ncbi.nlm.nih.gov/geo/query/acc.cgi?acc=GSE76737

GSE61298 https://www.ncbi.nlm.nih.gov/geo/query/acc.cgi?acc=GSE61298

GSE5099 https://www.ncbi.nlm.nih.gov/geo/query/acc.cgi?acc=GSE5099

GSE55536 https://www.ncbi.nlm.nih.gov/geo/query/acc.cgi?acc=GSE55536

GSE86298 https://www.ncbi.nlm.nih.gov/geo/query/acc.cgi?acc=GSE86298

GSE18686 https://www.ncbi.nlm.nih.gov/geo/query/acc.cgi?acc=GSE18686

GSE57614 https://www.ncbi.nlm.nih.gov/geo/query/acc.cgi?acc=GSE57614

GSE30595 https://www.ncbi.nlm.nih.gov/geo/query/acc.cgi?acc=GSE30595

GSE36537 https://www.ncbi.nlm.nih.gov/geo/query/acc.cgi?acc=GSE36537

GSE69607 https://www.ncbi.nlm.nih.gov/geo/query/acc.cgi?acc=GSE69607

GSE106706 https://www.ncbi.nlm.nih.gov/geo/query/acc.cgi?acc=GSE106706

## Ethics Statement

The studies involving human participants were reviewed and approved by the Institutional Ethics Committee of Zhongda Hospital. The patients/participants provided their written informed consent to participate in this study. The animal study was reviewed and approved by The Committee of Animal Care and Use of Southeast University. Written informed consent was obtained from the owners for the participation of their animals in this study.

## Author Contributions

SZ had full access to all of the data in the study and took responsibility for the integrity and accuracy of the data analysis. SZ and CC performed the data download, bioinformatic analysis, experiments, and preparation of the article for publication. All authors participated in writing the article and preparing the figures All authors contributed to the article and approved the submitted version.

## Funding

Supported in part by grants from the National Natural Science Foundation of China (grant numbers: 81571847, 81930058), the projects of Jiangsu Provincial Medical Key Discipline (ZDXKA2016025) and Jiangsu Provincial Special Program of Medical Science (BE2018743).

## Conflict of Interest

The authors declare that the research was conducted in the absence of any commercial or financial relationships that could be construed as a potential conflict of interest.
