# Supplementary material for: IFIH1 Contributes to M1 Macrophage Polarization in ARDS
Source: Front Immunol. 2021 Jan 14;11:580838. doi: 10.3389/fimmu.2020.580838 (PMC7841399; doi:10.3389/fimmu.2020.580838)
Supplement: Supplementary file 1 [file DataSheet_1.doc]

Catalogue of Supplemental Material

| ***Title*** | ***Content*** | ***Page*** |
| --- | --- | --- |
| **Figure S1** | **The flow chart to screen available mRNA datasets of macrophage M1 polarization in GEO database** | 2 |
| **Figure S2** | **External datasets validate that the 5 hub genes may be involved in M1 macrophage polarization** | 3 |
| **Figure S3** | **Immunofluorescence indicated that IFIH1 protein was significantly up-regulated in M1-polarized macrophage** | 6 |
| **Figure S4** | **50ng/mL was the optimum concentration for Poly(I:C) derived M1-macrophage** | 7 |
| **Figure S5** | **The knock-down efficacy of transfection in RAW264.7** | 8 |
| **Figure S6** | **The over-express efficacy of transfection in RAW264.7** | 9 |
| **Figure S7** | **Isolation, culture and identification of BMDMs** | 10 |
| **Figure S8** | **The knock-down and over-express efficacy of transfection in BMDMs** | 11 |
| **Figure S9** | **GSEA predicted that IFIH1 regulated macrophage M1 polarization via the RIG-I pathway** | 12 |
| **Table S1** | **ARDS patients' demographic and clinical characteristics** | 13 |
| **Table S2** | **The mRNA primers in this study for RT-PCR** | 14 |
| **Table S3** | **3 different sequences specially targeted to mouse IFIH1** | 15 |
| **Table S4** | **The sequences of short-hairpin RNAs** | 16 |
| **Table S5** | **The high-throughput experiments included in this study** | 17 |
| **Table S6** | **Clinical information of patients from human samples experiments** | 18 |

SM= Supplemental Material


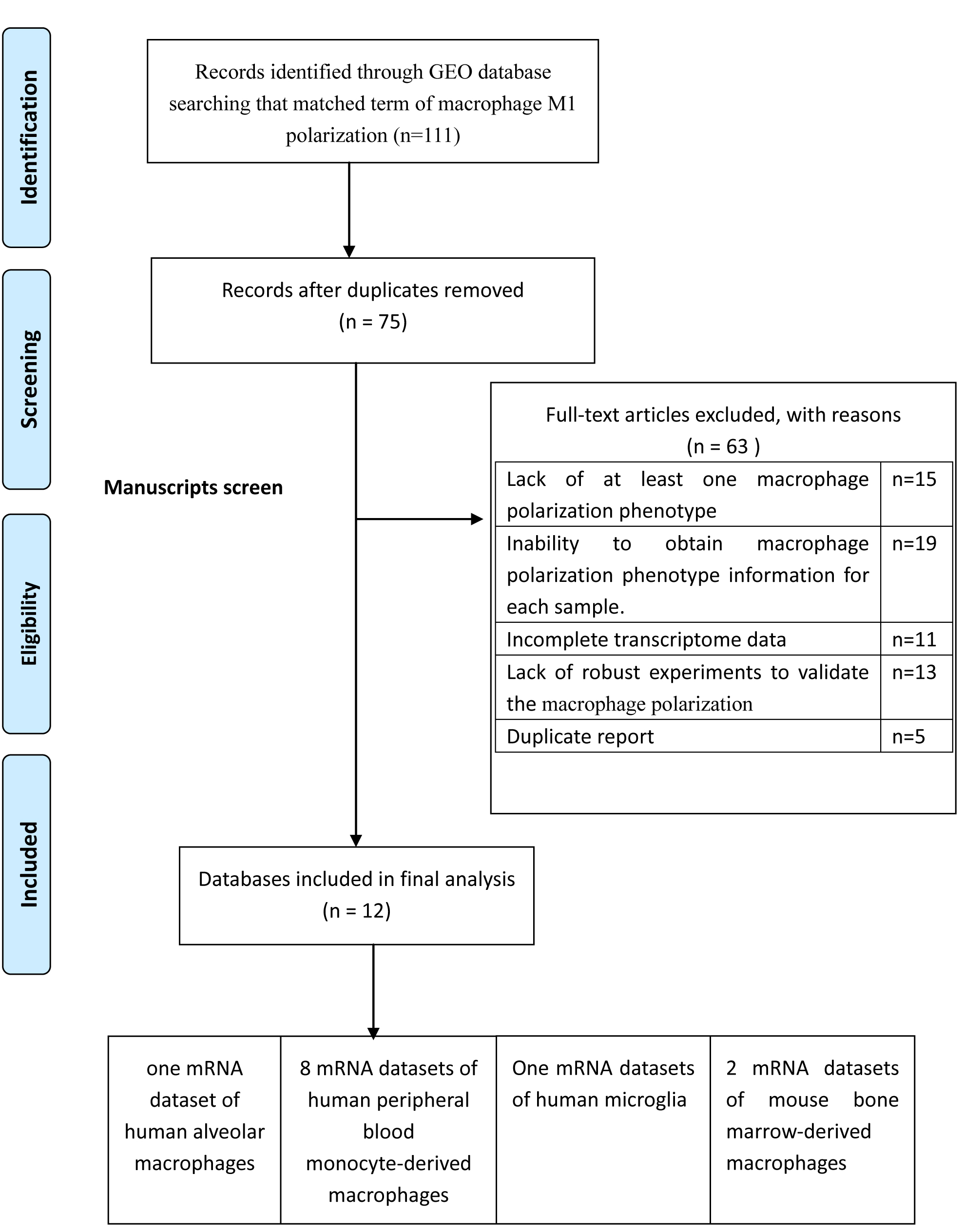


**Figure S1. The flow chart to screen available mRNA datasets of macrophage M1 polarization in GEO database**


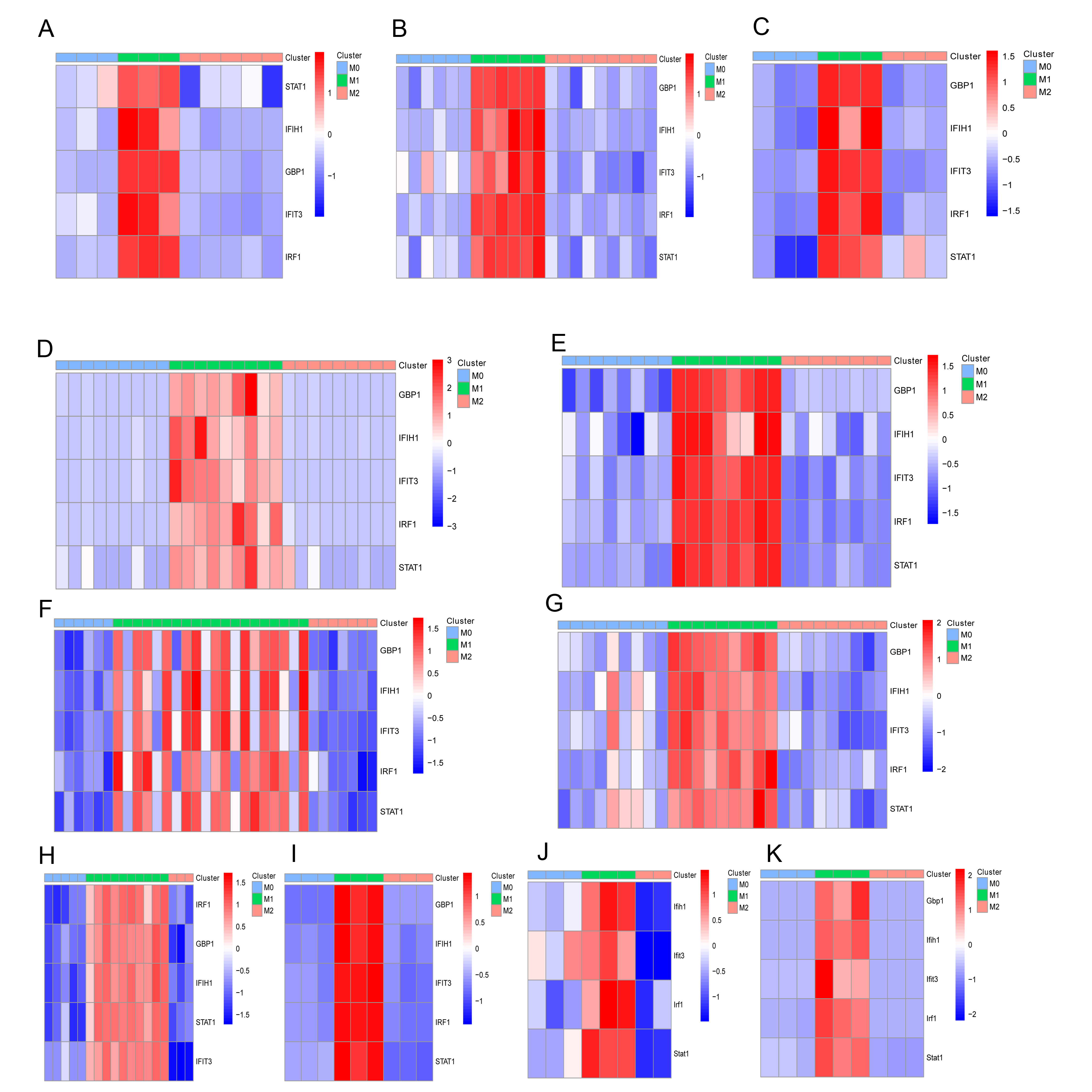


**Figure S2. External datasets validate that the 5 hub genes may be involved in M1 macrophage polarization**

a The heatmap of expression profiles from an external dataset (GSE76737, human human microglia-derived macrophages) validated that the 5 hub genes were significantly upregulated in M1-polarized macrophages *(FDR*<0.05).

b The heatmap of expression profiles from an external dataset (GSE61298, human peripheral blood monocyte-derived macrophages) validated that the 5 hub genes were significantly upregulated in M1-polarized macrophages *(FDR*<0.05).

c The heatmap of expression profiles from an external dataset (GSE5099, human peripheral blood monocyte-derived macrophages) validated that the 5 hub genes were significantly upregulated in M1-polarized macrophages *(FDR*<0.05).

d The heatmap of expression profiles from an external dataset (GSE55536, human peripheral blood monocyte-derived macrophages) validated that the 5 hub genes were significantly upregulated in M1-polarized macrophages (*FDR*<0.05).

e The heatmap of expression profiles from an external dataset (GSE86298, human peripheral blood monocyte-derived macrophages) validated that the 5 hub genes were significantly upregulated in M1-polarized macrophages (*FDR*<0.05).

f The heatmap of expression profiles from an external dataset (GSE18686, human peripheral blood monocyte-derived macrophages) validated that the 5 hub genes were significantly upregulated in M1-polarized macrophages (*FDR*<0.05).

g The heatmap of expression profiles from an external dataset (GSE57614, human peripheral blood monocyte-derived macrophages) validated that the 5 hub genes were significantly upregulated in M1-polarized macrophages (*FDR*<0.05).

h The heatmap of expression profiles from an external dataset (GSE30595, human peripheral blood monocyte-derived macrophages) validated that the 5 hub genes were significantly upregulated in M1-polarized macrophages (*FDR*<0.05).

i The heatmap of expression profiles from an external dataset (GSE36537, human peripheral blood monocyte-derived macrophages) validated that the 5 hub genes were significantly upregulated in M1-polarized macrophages (*FDR*<0.05).

j The heatmap of expression profiles from an external dataset (GSE69607, mouse bone marrow-derived macrophages) validated that the 5 hub genes were significantly upregulated in M1-polarized macrophages (*FDR*<0.05).

k The heatmap of expression profiles from an external dataset (GSE106706, mouse bone marrow-derived macrophages) validated that the 5 hub genes were significantly upregulated in M1-polarized macrophages (*FDR*<0.05).


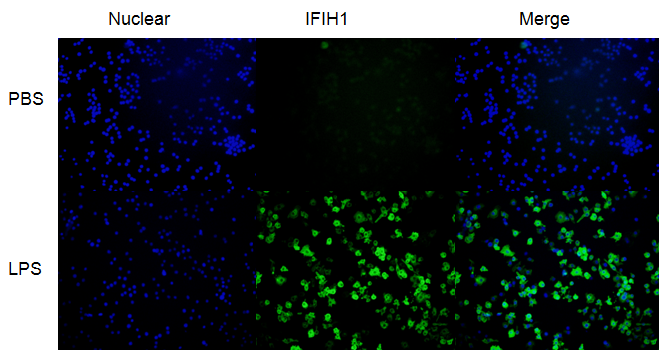


**Figure S3. Immunofluorescence indicated that IFIH1 protein was significantly up-regulated in M1-polarized macrophage**


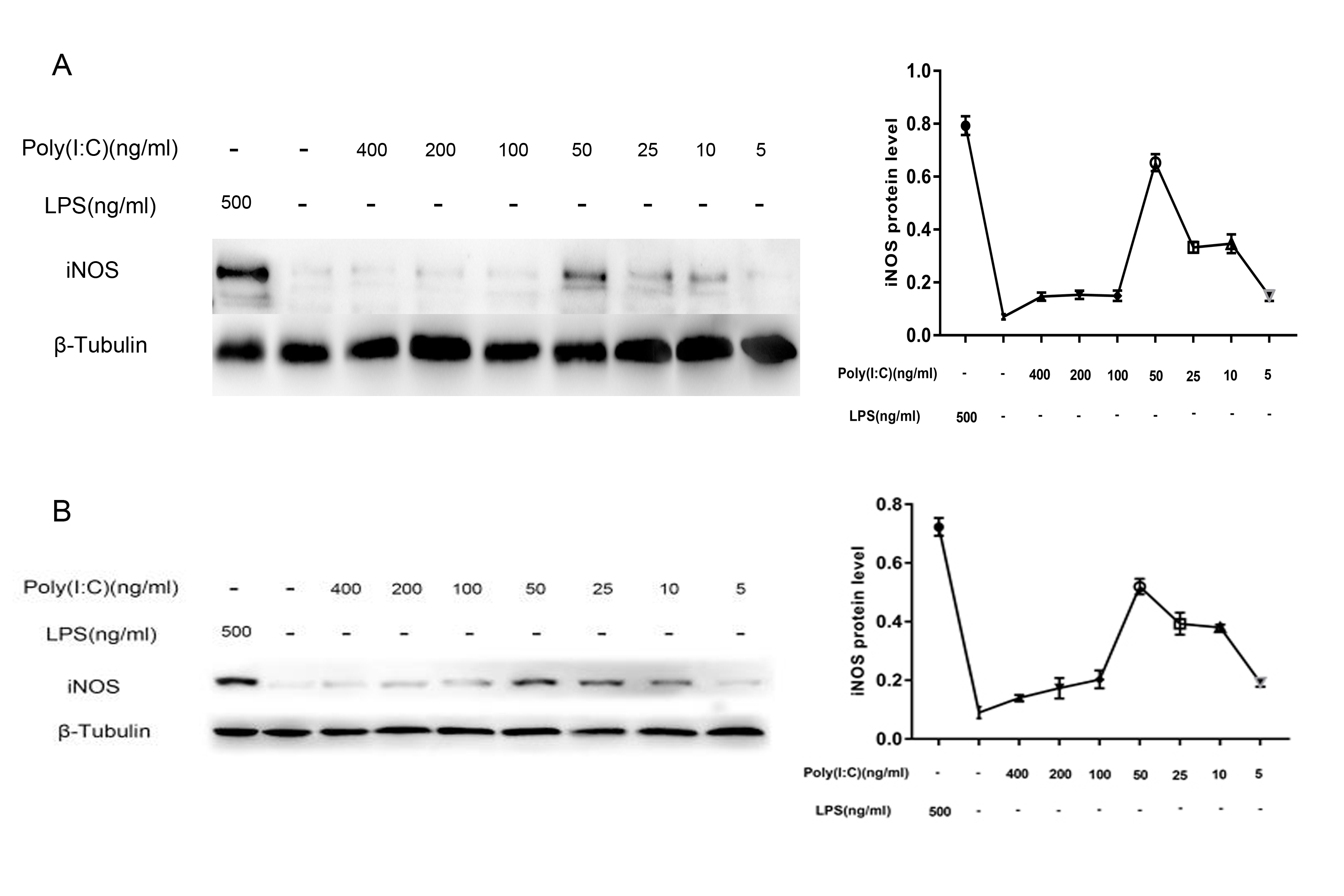


**Figure S4. 50ng/mL was the optimum concentration for Poly(I:C) derived M1-macrophage**

A. Western blot indicated that 0ng/mL was the optimum concentration for Poly(I:C) derived M1-macrophage in RAW264.7.

B. Western blot indicated that 50ng/mL was the optimum concentration for Poly(I:C) derived M1-macrophage in BMDMs.


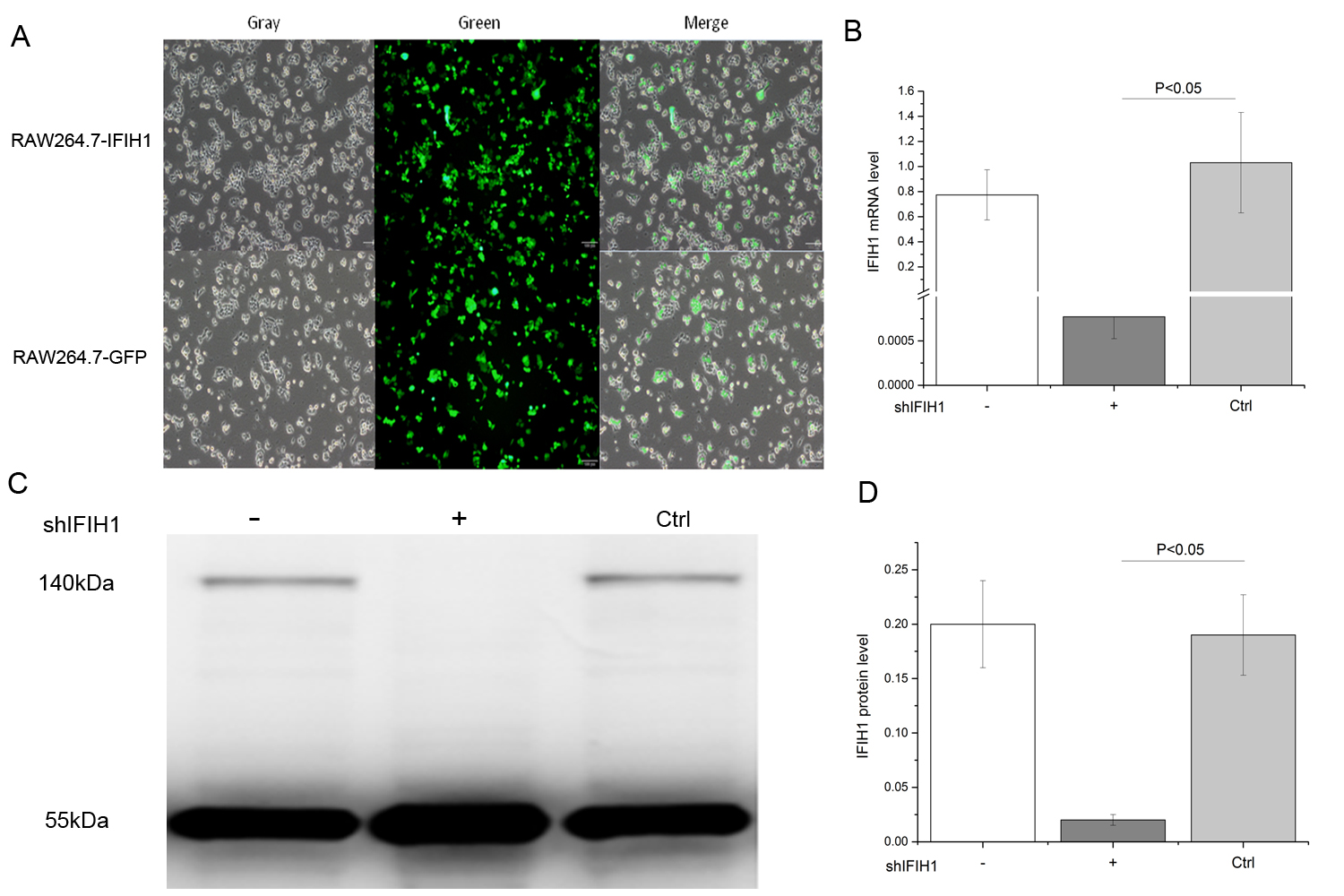


**Figure S5. The knock-down efficacy of transfection in RAW264.7**

a The efficiency of transfection in shRNAs against IFIH1 and control group in RAW264.7 cells.

b The knock-down efficiency of shRNAs against IFIH1 in RAW264.7 cells validated by qRT-PCR. The statistical data are from three independent experiments, and the bar indicates the SD values.

c The knock-down efficiency of shRNAs against IFIH1 in RAW264.7 cells validated by western blot.

d Quantitative analysis of c. The statistical data are from three independent experiments, and the bar indicates the SD values.


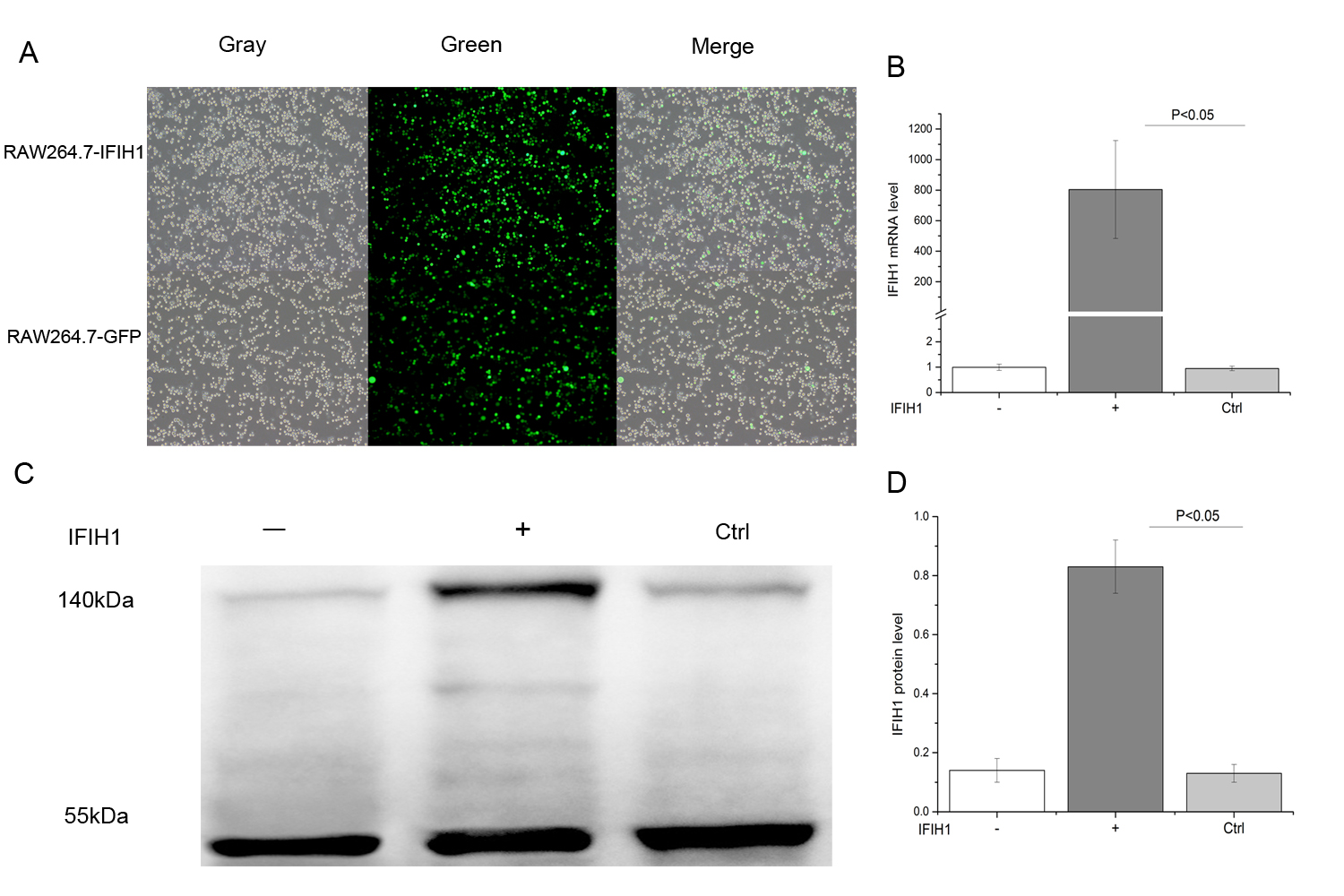


**Figure S6. The over-express efficacy of transfection in RAW264.7**

a The efficiency of transfection in plasmid vectors of IFIH1 and blank plasmid in RAW264.7 cells.

b The over-express efficiency of plasmid vectors in RAW264.7 cells validated by qRT-PCR. The statistical data are from three independent experiments, and the bar indicates the SD values.

c The over-express efficiency of plasmid vectors in RAW264.7 cells validated by western blot.

d Quantitative analysis of c. The statistical data are from three independent experiments, and the bar indicates the SD values.


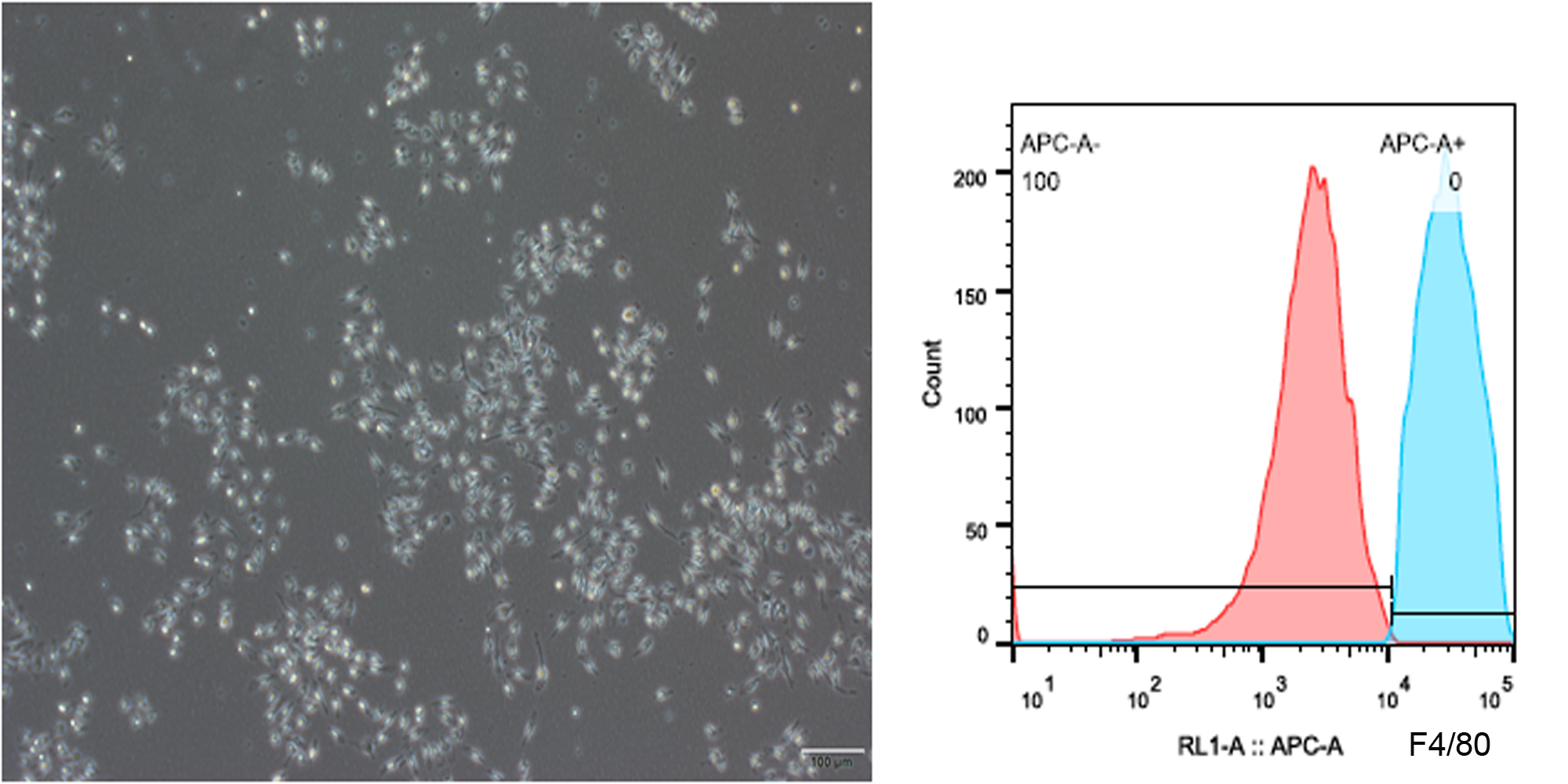


**Figure S7. Isolation, culture and identification of bone marrow- derived macrophages (BMDMs)**


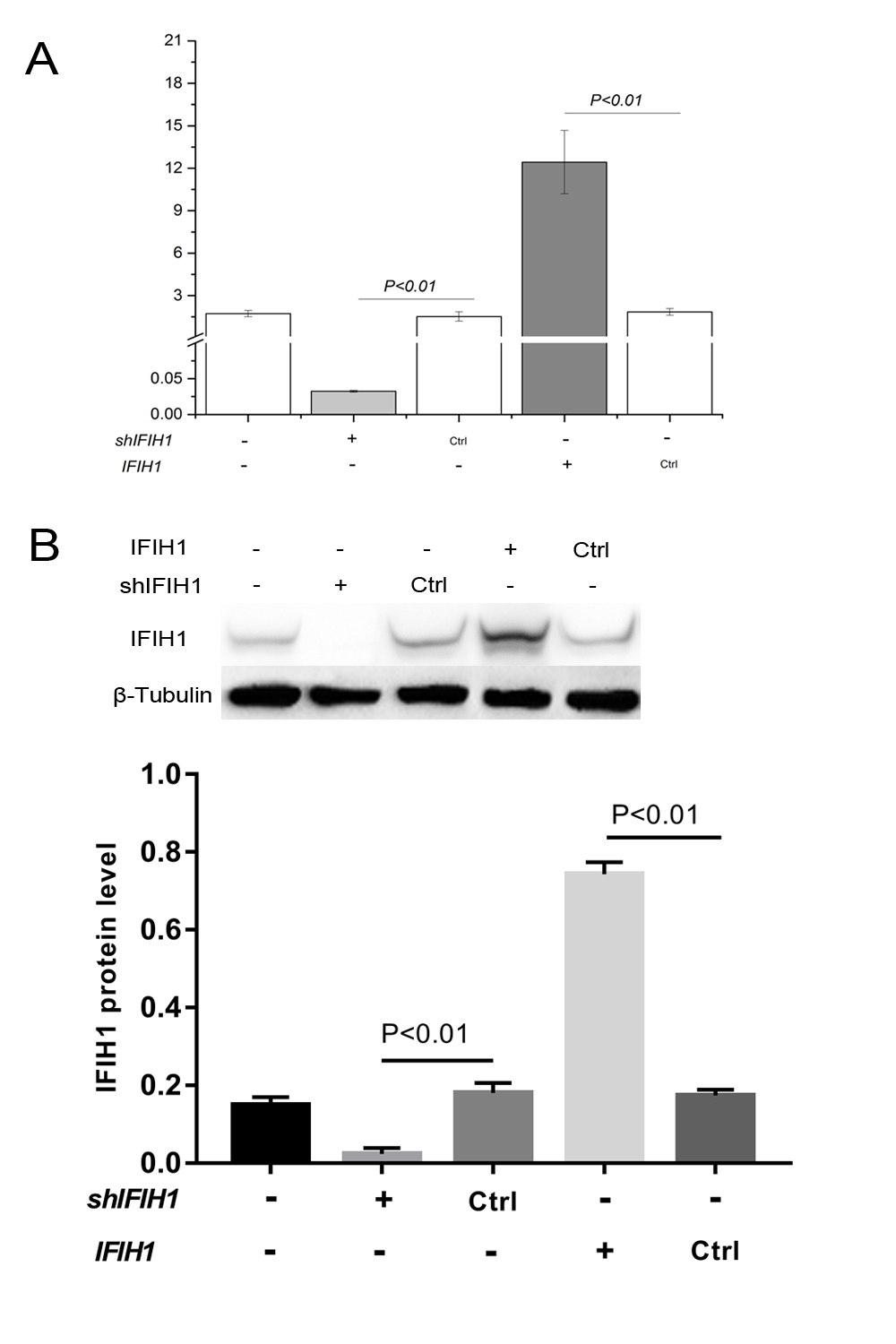


**Figure S8. The knock-down and over-express efficacy of transfection in BMDMs**

a The over-express efficiency of shIFIH1 and IFIH1 plasmid vectors in BMDMs validated by qRT-PCR. The statistical data are from three independent experiments, and the bar indicates the SD values.

b The over-express efficiency of shIFIH1 and IFIH1plasmid vectors in BMDMs validated by western blot, with quantitative analysis. The statistical data are from three independent experiments, and the bar indicates the SD values.


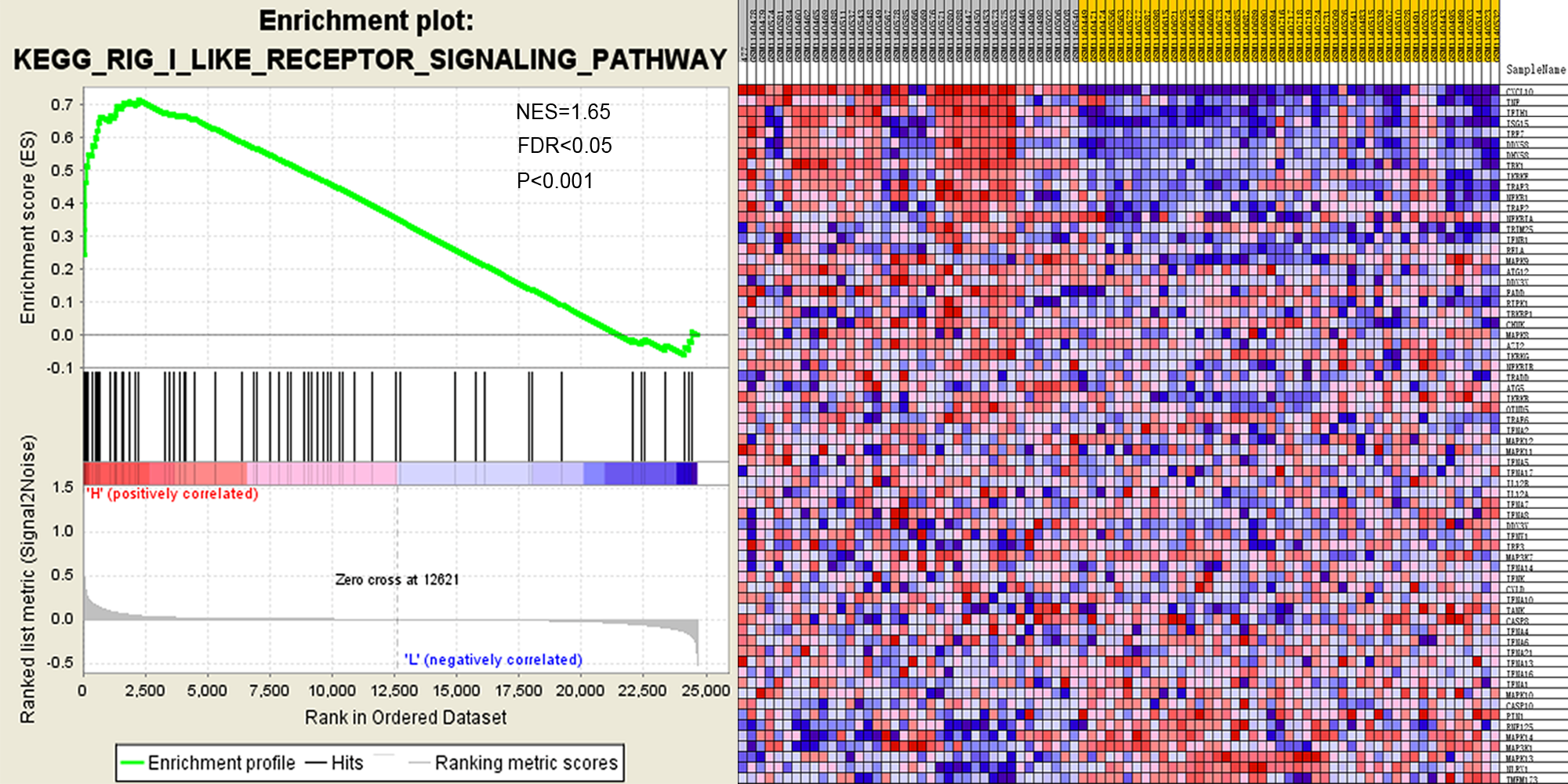


**Figure S9. GSEA predicted that IFIH1 regulated macrophage M1 polarization via the RIG-I pathway**

GSEA indicated that the RIG-I pathway was significantly activated and genes in the RIG-I pathway were obviously enriched when IFIH1 expression was upregulated (*FDR*<0.05).

Table S1. 26 ARDS patients' demographic and clinical characteristics

| **Patient ID** | **Age** | **Gender** | **Severity of ARDS** | **APACHE II** |
| --- | --- | --- | --- | --- |
| **1** | 52 | Female | 3 | 20 |
| **2** | 44 | Male | 1 | 25 |
| **3** | 63 | Male | 1 | 18 |
| **4** | 42 | Male | 3 | 19 |
| **5** | 54 | Female | 3 | 12 |
| **6** | 60 | Female | 1 | 24 |
| **7** | 24 | Female | 3 | 25 |
| **8** | 63 | Male | 2 | 10 |
| **9** | 57 | Male | 2 | 13 |
| **10** | 51 | Male | 2 | 19 |
| **11** | 40 | Male | 2 | 18 |
| **12** | 62 | Female | 2 | 23 |
| **13** | 50 | Male | 2 | 11 |
| **14** | 19 | Male | 3 | 29 |
| **15** | 56 | Female | 1 | 9 |
| **16** | 33 | Female | 1 | 29 |
| **17** | 52 | Male | 1 | 25 |
| **18** | 68 | Female | 2 | 20 |
| **19** | 37 | Male | 3 | 30 |
| **20** | 24 | Male | 3 | 24 |
| **21** | 34 | Male | 3 | 30 |
| **22** | 65 | Male | 1 | 26 |
| **23** | 21 | Male | 3 | 26 |
| **24** | 56 | Female | 1 | 17 |
| **25** | 54 | Male | 3 | 33 |
| **26** | 66 | Male | 1 | 13 |

Table S2. The mRNA primers in this study for RT-PCR

| **species** | **Gene** | **Forward primer** | **Reverse primer** |
| --- | --- | --- | --- |
| **Mus** | Stat1 | TCACAGTGGTTCGAGCTTCAG | CGAGACATCATAGGCAGCGTG |
| **Mus** | Ifih1 | GTGATGACGAGGCCAGCAGTTG | ATTCATCCGTTTCGTCCAGTTTCA |
| **Mus** | Irf1 | ATGCCAATCACTCGAATGCG | TTGTATCGGCCTGTGTGAATG |
| **Mus** | Ifit3 | GAGGACAACCGGAAGTGTGT | GGATGAGCAGAGGAGTCAGG |
| **Mus** | Gbp1 | AAGAAGTGAAGCAGGGGACA | GCGGCTTCTGCTTTTATACG |
| **Mus** | IFN-β | AGCCAGGAGCTTGAATAAAATGAATATTAG | CTGGTGTCGGCTGCTACCTGCAAGATGAG |
| **Mus** | β-actin | TTGCTGACAGGATGCAGAAG | ACTCCTGCTTGCTGATCCACAT |
| **Homo** | STAT1 | GAACTTTCTGCTGTTACTTTCCCTG | TTGGCTCTGGTGCTTCCTTTG |
| **Homo** | IFIH1 | GCCACAAAAAATCACAAGCCA | TCCATTGTCTGGATTTAAGCGG |
| **Homo** | IRF1 | AAGGATGCCTGTTTGTTCCG | CAGCGAAAGTTGGCCTTCC |
| **Homo** | IFIT3 | GCTGATGGGATTGTTGCT | CAGATTGGGTGCTGCTAC |
| **Homo** | GBP1 | TCACTTCGTTTCCTCCGAGAT | CCCTCGCACACAAGCACAGTA |
| **Homo** | β-actin | AGCGAGCATCCCCCAAAGTT | GGGCACGAAGGCTCATCATT |

Table S3. 3 different sequences specially targeted to mouse IFIH1

| **ID** | **Start** | **Target sequence (DNA)** | **GC%** |
| --- | --- | --- | --- |
| **1** | 423 | GGGACTATTGACAGTCGAAGA | 47.62 |
| **2** | 774 | GCAGCTCAGGCCTTACCAAAT | 52.39 |
| **3** | 1245 | GCAGAAGCTGAGAAACAATGA | 42.86 |

Table S4. The sequences of short-hairpin RNAs

| **ID** | **name** | **Oligo Sequence** |
| --- | --- | --- |
| **1** | i1-F | gatccGGGACTATTGACAGTCGAAGATCAAGAGTCTTCGACTGTCAATAGTCCCtttttt |
|  | i1-R | aattaaaaaaGGGACTATTGACAGTCGAAGACTCTTGATCTTCGACTGTCAATAGTCCCg |
| **2** | i2-F | gatccGCAGCTCAGGCCTTACCAAATTCAAGAGATTTGGTAAGGCCTGAGCTGCtttttt |
|  | i2-R | aattaaaaaaGCAGCTCAGGCCTTACCAAATCTCTTGAATTTGGTAAGGCCTGAGCTGCg |
| **3** | i3-F | gatccGCAGAAGCTGAGAAACAATGATCAAGAGTCATTGTTTCTCAGCTTCTGCtttttt |
|  | i3-R | aattaaaaaaGCAGAAGCTGAGAAACAATGACTCTTGATCATTGTTTCTCAGCTTCTGCg |

Table S5. The high-throughput experiments included in this study

| **ID** | **Species** | **Source** | **Country** | **M0 sample size** | **M1 sample size** | **M2 sample size** |
| --- | --- | --- | --- | --- | --- | --- |
| **GSE46903** | Homo | Alveolar  macrophage | Germany | 47 | 38 | 40 |
| **GSE76737** | Homo | Microglia | Canada | 3 | 3 | 6 |
| **GSE61298** | Homo | Monocyte macrophage | Austria | 6 | 6 | 9 |
| **GSE5099** | Homo | Monocyte macrophage | Britain | 3 | 3 | 3 |
| **GSE55536** | Homo | Monocyte macrophage | USA | 9 | 9 | 9 |
| **GSE86298** | Homo | Monocyte macrophage | Germany | 8 | 8 | 8 |
| **GSE18686** | Homo | Monocyte macrophage | USA | 6 | 20 | 7 |
| **GSE57614** | Homo | Monocyte macrophage | Italy | 9 | 9 | 9 |
| **GSE30595** | Homo | Monocyte macrophage | Italy | 6 | 9 | 3 |
| **GSE36537** | Homo | Monocyte macrophage | France | 3 | 3 | 3 |
| **GSE69607** | Mus | Monocyte macrophage | USA | 3 | 3 | 2 |
| **GSE106706** | Mus | Monocyte macrophage | Hungary | 3 | 3 | 3 |

Table S6. Clinical information of patients from human samples experiments

| **Patients' ID** | **Age** | **Male** | **Apache II score** | **SOFA score** | **Diagnosis** |
| --- | --- | --- | --- | --- | --- |
| **ARDS 1** | 72 | Male | 39 | 11 | 1. pneumonia, ARDS (moderate);  2. hypertension (3 level, very high risk) |
| **ARDS 2** | 78 | Female | 12 | 7 | 1. pneumonia, ARDS (severe);  2. hypertension (3 level, very high risk) |
| **ARDS 3** | 60 | Male | 12 | 8 | 1. pneumonia, ARDS (severe);  2. sepsis, septic shock |
| **ARDS 4** | 55 | Male | 11 | 1 | 1. pneumonia, ARDS (moderate); |
| **ARDS 5** | 53 | Male | 18 | 7 | 1. pneumonia, ARDS (severe);  2. sepsis, septic shock |
| **ARDS 6** | 65 | Female | 19 | 8 | 1. pneumonia, ARDS (severe);  2. sepsis, septic shock |
| **Control 1** | 53 | Male | 18 | 8 | 1. cerebral hemorrhage, cerebral hernia  2. aortic dissection  3. hypertension (3 level, very high risk) |
| **Control 2** | 39 | Male | 14 | 7 | 1. basal ganglia cerebral hemorrhage,  cerebral hernia  2. hypertension (3 level, very high risk) |
| **Control 3** | 69 | Male | 19 | 9 | 1. acute cerebral infarction  2. hypertension (3 level, very high risk) |
| **Control 4** | 48 | Male | 19 | 4 | 1. epidural hematoma  2. hypertension (3 level, very high risk) |
| **Control 5** | 45 | Female | 24 | 7 | 1. thalamic hemorrhage |
| **Control 6** | 51 | Female | 17 | 7 | 1. cerebral hemorrhage, cerebral hernia  2. hypertension (3 level, very high risk) |
